# Mapping the intrinsic photocurrent streamlines through micromagnetic heterostructure devices

**DOI:** 10.1073/pnas.2221815120

**Published:** 2023-09-18

**Authors:** David Mayes, Farima Farahmand, Maxwell Grossnickle, Mark Lohmann, Mohammed Aldosary, Junxue Li, Vivek Aji, Jing Shi, Justin C. W. Song, Nathaniel M. Gabor

**Affiliations:** ^a^Department of Physics and Astronomy, University of California, Riverside, CA 92521; ^b^Laboratory of Quantum Materials Optoelectronics, University of California, Riverside, CA 92521; ^c^Division of Physics and Applied Physics, School of Physical and Mathematical Sciences, Nanyang Technological University, Singapore 637371, Singapore

**Keywords:** photocurrent, magnetic devices, optoelectronics, physics

## Abstract

Photocurrent in quantum materials is often collected at global contacts far away from the initial photoexcitation. This collection process is highly nonlocal. It involves an intricate spatial pattern of photocurrent flow (streamlines) away from its primary photoexcitation that depends sensitively on the configuration of current collecting contacts as well as the spatial nonuniformity and tensor structure of conductivity. Direct imaging to track photocurrent streamlines is challenging. Here, we demonstrate a microscopy method to image photocurrent streamlines through ultrathin heterostructure devices comprising platinum on yttrium iron garnet (YIG). We accomplish this by combining scanning photovoltage microscopy with a uniform rotating magnetic field. Here, local photocurrent is generated through a photo-Nernst type effect with its direction controlled by the external magnetic field. This enables the mapping of photocurrent streamlines in a variety of geometries that include conventional Hall bar-type devices, but also unconventional wing-shaped devices called electrofoils. In these, we find that photocurrent streamlines display contortion, compression, and expansion behavior depending on the shape and angle of attack of the electrofoil devices, much in the same way as tracers in a wind tunnel map the flow of air around an aerodynamic airfoil. This affords a powerful tool to visualize and characterize charge flow in optoelectronic devices.

## Significance

Direct imaging to track photocurrent streamlines in quantum optoelectronic devices remains a key challenge in understanding exotic device behavior. Here, we combine laser imaging with light-sensitive devices to show images of photocurrent streamlines through a working device. We design a type of device called an electrofoil that allows us to contort, compress, and expand these photocurrent streamlines in a similar way to wings, which contort, compress, and expand the flow of air. With imaging resolution spanning over an order of magnitude in spatial scale, we have shown that photocurrent streamline microscopy is a robust experimental tool for obtaining detailed visualization of photocurrent in quantum materials.

Photocurrent in optoelectronic devices often adopts a long-range character: Photocurrent generation at a spatially localized laser spot can induce voltage/current signals at contacts far away, even in nonballistic electronic materials. This remote sensing of photocurrent yields a wealth of complex spatial photocurrent maps that enable the identification of local symmetry breaking (1), chirality (2), and electronic behavior in topological surface states (3, 4). The patterns are predicted to obey the Shockley–Ramo theorem (5, 6)for conductors (7), wherein a local electromotive force creates a global diffusion current that flows into the device contacts. For local charge photocurrent density $\vec{J_{c}}$ generated at a local laser spot, the global photovoltage $V_{\mathrm{ph}}$,

[1] $$\begin{array}{ccc} V_{ph} & = & R_{G}\int \vec{J_{c}}\left(\vec{r}\right)\cdot \vec{S}\left(\vec{r}\right)d^{2}\vec{r}, \end{array}$$

is highly sensitive to the smoothly varying streamline pattern $\vec{S}\left(\vec{r}\right)$, which depends on both *local* properties (conductivity) and *global* boundaries of the system. In Eq. **1**, the photocurrent streamline $\vec{S}\left(\vec{r}\right)$ describes a spatially varying directional pattern along which spatially local photocurrent generates maximum photoinduced voltage when collected at far-away contacts; *R*_G_ is the configuration-specific resistance determined by the measurement geometry. Eq. **1** is agnostic to the precise mechanism for photocurrent generation and has been applied to diverse settings that range from bulk quantum geometric photocurrent generation (1, 8) to those of the thermoelectric/Nernst origin (2, 9).

In the investigation of spatially resolved photoresponse, photocurrent streamline fields $\vec{S}\left(\vec{r}\right)$ are typically numerically calculated [by solving a suitable Laplace boundary value problem (7)] and are used to phenomenologically explain photocurrent spatial maps (1–4, 10). However, direct empirical visualization of $\vec{S}\left(\vec{r}\right)$ has remained challenging as it requires precise and continuous control over the direction of local photocurrent density $\vec{j_{c}}\left(\vec{r}\right)$. In particular, generating a local photocurrent often requires constructing in-plane p-n junctions that inevitably influence the photocurrent streamline field $\vec{S}\left(\vec{r}\right)$. Further, photocurrents at p-n junctions often have a direction that is locked at the device fabrication stage; its direction cannot be controlled in situ, thus limiting the ability to probe the full vector field $\vec{S}\left(\vec{r}\right)$. Last, while several techniques exist that can probe electronic current in nanoscale devices, including scanning single electron transistors (11, 12), magnetometers (13–16), and quantum gases (17, 18), these local measurement techniques do not track how local photocurrent generation translates to global device characteristics. While theoretical modeling has been broadly applied (3, 7, 10, 19, 20), experimental techniques that actively control directional current flow while simultaneously imaging the global device response and photocurrent streamlines must be developed in order to advance our understanding of complex optoelectronic device behavior.

Here, we report direct empirical imaging of the photocurrent streamline $\vec{S}\left(\vec{r}\right)$. This is enabled by in situ control of the locally generated photocurrent density in a micromagnetic heterostructure, Pt thin film on YIG substrate (Pt/YIG). Our photocurrent streamline microscopy technique, described below, exploits a photoinduced Nernst-type effect in these micromagnetic heterostructures. In particular, by using a focused laser beam, we induce an *out-of-plane* temperature gradient across the Pt/YIG interface; a Nernst-type effect across the interface enables to generate an *in-plane* local photocurrent with a direction controllable by an in-plane magnetic field and a position (spatial resolution) controlled by the laser spot. This technique overcomes the need for (and challenge of) in-plane p-n junctions by exploiting the out-of-plane Pt/YIG interface and a transverse Nernst-type current. We use this technique for a full empirical mapping of the photocurrent streamlines in a wide variety of device configurations and demonstrate how photocurrent streamlines depend sensitively on device geometry and can display a wide variety of contortion, compression, and expansion behavior.

**Fig. 1.**
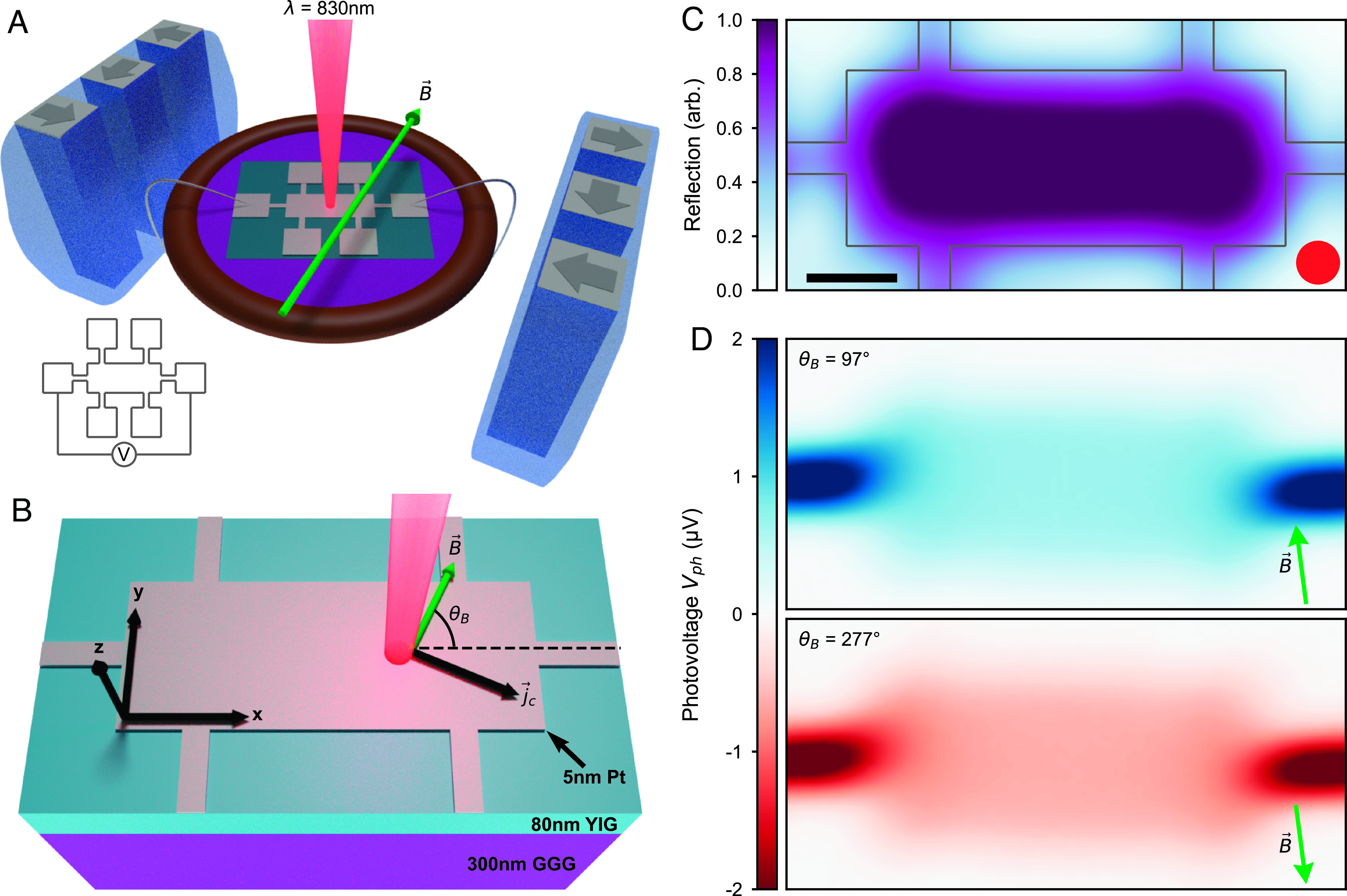
Photocurrent streamline microscopy of micromagnetic heterostructure devices. (*A*) Schematic of the magnetic heterostructure Hall bar device and measurement. The green arrow indicates the magnetic field direction. *Inset*, two-terminal photovoltage configuration; all side contacts are grounded. (*B*) Schematic of Device 1, a conventional Hall bar defined in ultrathin Pt (5 nm thick) sitting atop a ferrimagnetic layer of YIG (80 nm) with a substrate of GGG (300 nm). Laser illumination *λ* = 830 nm; average power density 3.6 MW/cm^2^. Charge current $j_{c}$ is perpendicular to the applied magnetic field *B*. $\theta_{B}$ is the angle between the magnetic field, and the axis defined by the photovoltage probes (*x* axis). (*C*) Reflection map of Device 1. The red circle indicates the full width at half maximum (FWHM) of the beam spot. (Scale bar, 50 μ.) (*D*) Magnetic field-dependent photovoltage $V_{\mathrm{ph}}$ maps at $\theta_{B}$ = 97°, 277°. The *B*-field direction is indicated by green arrows. Magnetic field magnitude *B* = 0.52 T for all measurements.

Our imaging scheme builds on prior magneto-thermal photoexcitation techniques (21–26). Fig. 1 shows the scanning magneto-photovoltage microscopy (SMPM) technique developed here to study the photovoltage response in ultrathin micromagnetic devices. Utilizing a carefully structured Halbach array of permanent magnets, we establish a magnetic field $\vec{B}$ that can be rotated through the entire solid angle of three-dimensional space (Fig. 1*A*). In the uniform region of the *B*-field, a scanning laser (wavelength λ = 830 nm) is used to generate photoinduced voltage response within the metal–magnet heterostructure devices. The devices studied in this work, shown schematically in Fig. 1*B*, are composed of ultrathin platinum patterned on a ferrimagnetic insulator yttrium iron garnet (YIG) thin film, which has been epitaxially grown on gadolinium gallium garnet (GGG) (*SI Appendix*, section S1). As the laser scans over the device, we measure the photovoltage at each point, while simultaneously imaging the device using the back-reflected light intensity. Taking advantage of high image stability, we acquire a sequence of photovoltage images, each with a unique magnetic field orientation (*SI Appendix*, section S2).

We first characterized a Pt/YIG Hall bar device by measuring photovoltage as a function of laser position and in-plane magnetic field angle *θ*_*B*_. At a fixed *θ*_*B*_, we scan the laser across the device and generate maps of the reflected intensity (Fig. 1*C*) and magnetic field-dependent photovoltage $V_{\mathrm{ph}}$ (Fig. 1*D*) (*SI Appendix*, sections S3 and S4). As shown in Fig. 1 *D*, *Top*, when we set the magnetic field to be situated across the device (*θ*_*B*_ = 97°) relative to the voltage probes (positioned along the *x* axis labeled in Fig. 1*B*), we observe spatially uniform positive photovoltage that is enhanced near the contacts. When the magnetic field is rotated by 180°, the polarity of $V_{\mathrm{ph}}$ changes from positive to negative, yet exhibits otherwise similar spatial features (Fig. 1 *D*, *Bottom*).

The photovoltage images of Fig. 1*D* are readily described by a thermomagnetic photoresponse. Laser illumination produces an in-plane ($\vec{r}=(x,y)$) *local* charge current density

[2] $$\vec{j_{c}}=-\;N\nabla_{z}T\left(\vec{r},z\right)\;\times \;\vec{B},$$

where N is a Nernst-type coefficient that relates the (out-of-plane) temperature gradient to the in-plane photocurrent $\vec{j_{c}}$ (see *SI Appendix*, section S3 for a detailed microscopic description); this response is similar to that of the Photo-Nernst type response detailed in ref. 2. The local charge current $\vec{j_{c}}$ flows in the direction orthogonal to both the out-of-plane temperature gradient $\nabla_{z}T$ (established across the Pt/YIG interface) and the magnetic field $\vec{B}$, each of which can be controlled by our experiment. This local in-plane charge current in turn—together with the photocurrent streamlines in Eq. **1**—generates a photoinduced voltage across the device at the current-carrying contacts. Indeed, by switching the direction of $\vec{B}$, the direction of $\vec{j_{c}}$ flips and produces opposite signs of laser-induced photovoltage at the global contacts (Fig. 1*D*); we note parenthetically that $\vec{B}$ in Fig. 1*D* points perpendicular to the direction connecting the photovoltage contacts, as expected from Eq. **2**.

**Fig. 2.**
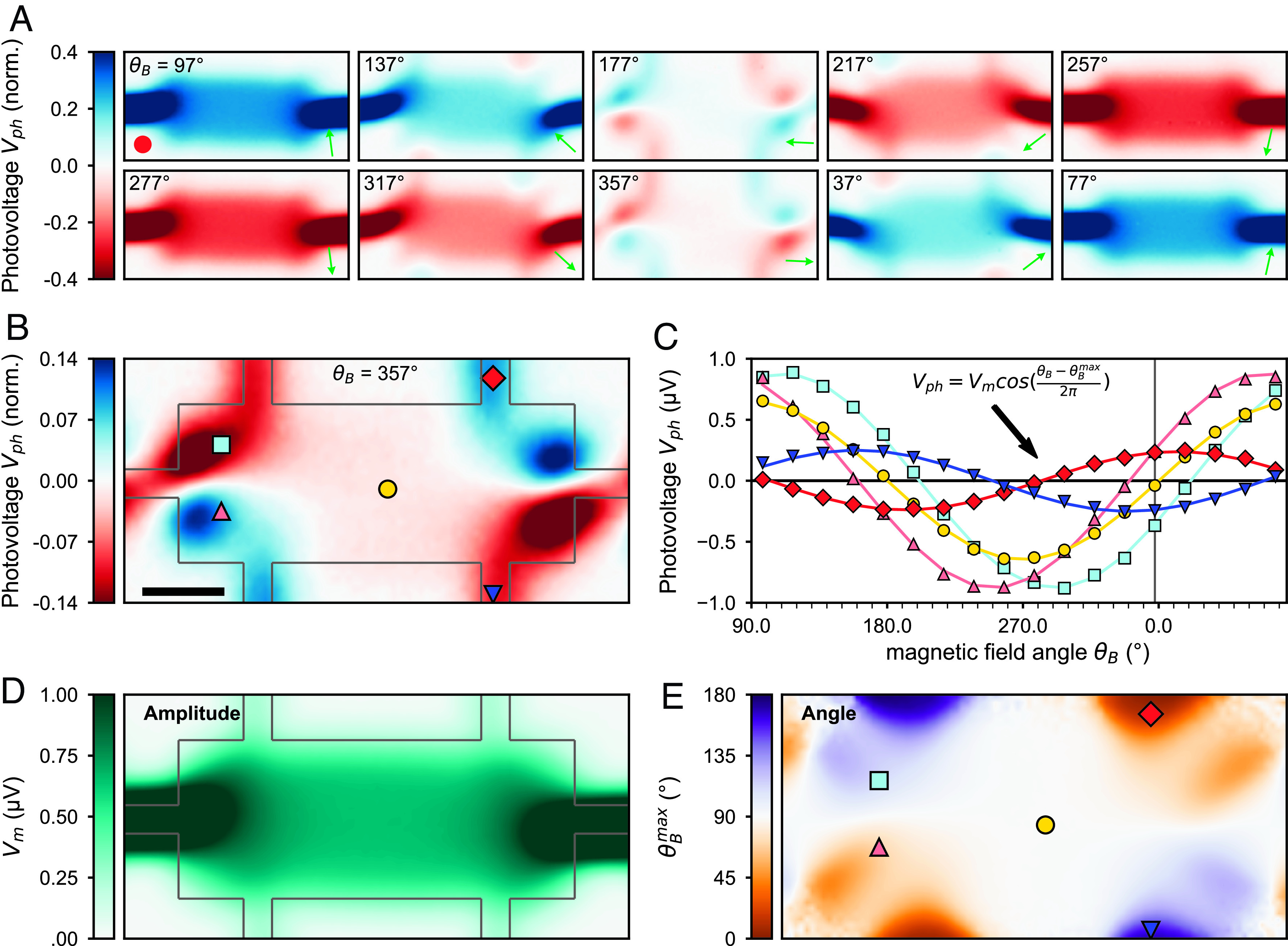
Imaging the magnetic field-dependent photovoltage in a micromagnetic heterostructure device. (*A*) Magnetic field–dependent photovoltage maps at 10 different magnetic field angles. *B*-field direction, $\theta_{B}$, is labeled top left corner and is indicated by green arrows. The red circle indicates beam spot size (FWHM = 27 µm). (*B*) Detailed image at $\theta_{B}$ = 357°. (Scale bar, 50 µ for all images.) (*C*) $V_{\mathrm{ph}}$ vs. $\theta_{B}$ at 5 different positions on the device, marked in *B*. Data points share the same colors and shapes as in *B*. At each position, $V_{\mathrm{ph}}$ vs. $\theta_{B}$ is fit to the function $V_{\mathrm{ph}}\left(\theta_{B}\right)=V_{m}\cos\left(\theta_{B}\;-\;\theta_{B}^{max}\right)$, shown as corresponding solid lines. (*D*) Image of the sinusoidal fit amplitude $V_{m}$ at all spatial positions. (*E*) Image of the angular phase shift of the fit $\theta_{B}^{\max}$ relative to $\theta_{B}$ = 0° at all spatial positions. Marked points are the same as those in *B*, corresponding to the $V_{\mathrm{ph}}$ vs. $\theta_{B}$ data in *C*.

The local in-plane photocurrent in Eq. **2** indicates an opportune experimental tool: Using a laser beam to generate a localized interfacial temperature gradient, we can control the direction of local charge current $\vec{j_{c}}$ by rotating the magnetic field angle *θ*_*B*_ (schematic Fig. 1*B*). Indeed, as shown in Fig. 2, optoelectronic measurements taken as *θ*_*B*_ was varied exhibit striking spatial patterns, with prominent photovoltage features emerging in corners and at narrow constrictions. These patterns are in sharp contrast to measurements taken with the in-plane magnetic field situated across the device, as in Fig. 1*D*. When the *B*-field is parallel to the voltage probe direction (*θ*_*B*_ = 177°, Fig. 2 *A*, *Middle* image), $V_{\mathrm{ph}}$ is suppressed in the central region of the device, as expected from ordinary thermomagnetic response. However, new spatial features appear at sharp corners and along transverse contacts. As the magnetic field rotates through 180°, the polarity of these anomalous features switches sign.

From scanning magneto-photovoltage images, we can examine in detail the evolution of the anomalous photovoltage features as a function of *θ*_*B*_. Fig. 2*B* shows a rescaled $V_{\mathrm{ph}}$ map measured at *θ*_*B*_ = 357°. For a point in the central region of the device (yellow circle Fig. 2*B*), we plot $V_{\mathrm{ph}}$ vs. *θ*_*B*_ in Fig. 2*C*. The data are well fitted by a sinusoidal function $V_{\mathrm{ph}}=V_{m}\cos\left(\theta_{B}-\theta_{B}^{\max}\right)$ that is characterized by two parameters: the photovoltage amplitude $V_{m}$ and the magnetic field angle at which $V_{\mathrm{ph}}$ reaches a maximum, $\theta_{B}^{\max}$. Consistent with Eq. **2**, the photovoltage in the central region (yellow data Fig. 2*C*) reaches a maximum of $V_{m}$ = 0.6 μV when *θ*_*B*_ is at a right angle to the *x* axis ($\theta_{B}^{\max}$ = 90°).

At different positions within the device, we find that maximum photovoltage results from a distinct alignment of the magnetic field, $\theta_{B}^{\max}$ (*x, y*). If the laser is fixed at the red diamond at the *Top* *Right* in Fig. 2 *B*, $V_{\mathrm{ph}}$ reaches a maximum when *θ*_*B*_ = 0°. This is in direct contrast to the blue triangle at the *Bottom* *Right* in Fig. 2 *B*, for which the photovoltage reaches a maximum when *θ*_*B*_ = 180°. When compared to the central region, both spatial features exhibit sinusoidal behavior that is offset by a 90° phase (Fig. 2*C*). To better visualize the variations of amplitude and phase patterns, we generate images of the photovoltage amplitude $V_{m}$ (*x, y*) and angular offset $\theta_{B}^{\max}$ (*x, y*) at *all* points in space using the sinusoidal fits in Fig. 2*C*. While the amplitude $V_{m}$ (*x, y*) (Fig. 2*D*) looks qualitatively similar to the ordinary photoresponse of Fig. 1, the angular offset $\theta_{B}^{\max}$ (*x, y*) exhibits a rich structure that gives the magnetic field angle required to maximize the photovoltage (Fig. 2*E*).

**Fig. 3.**
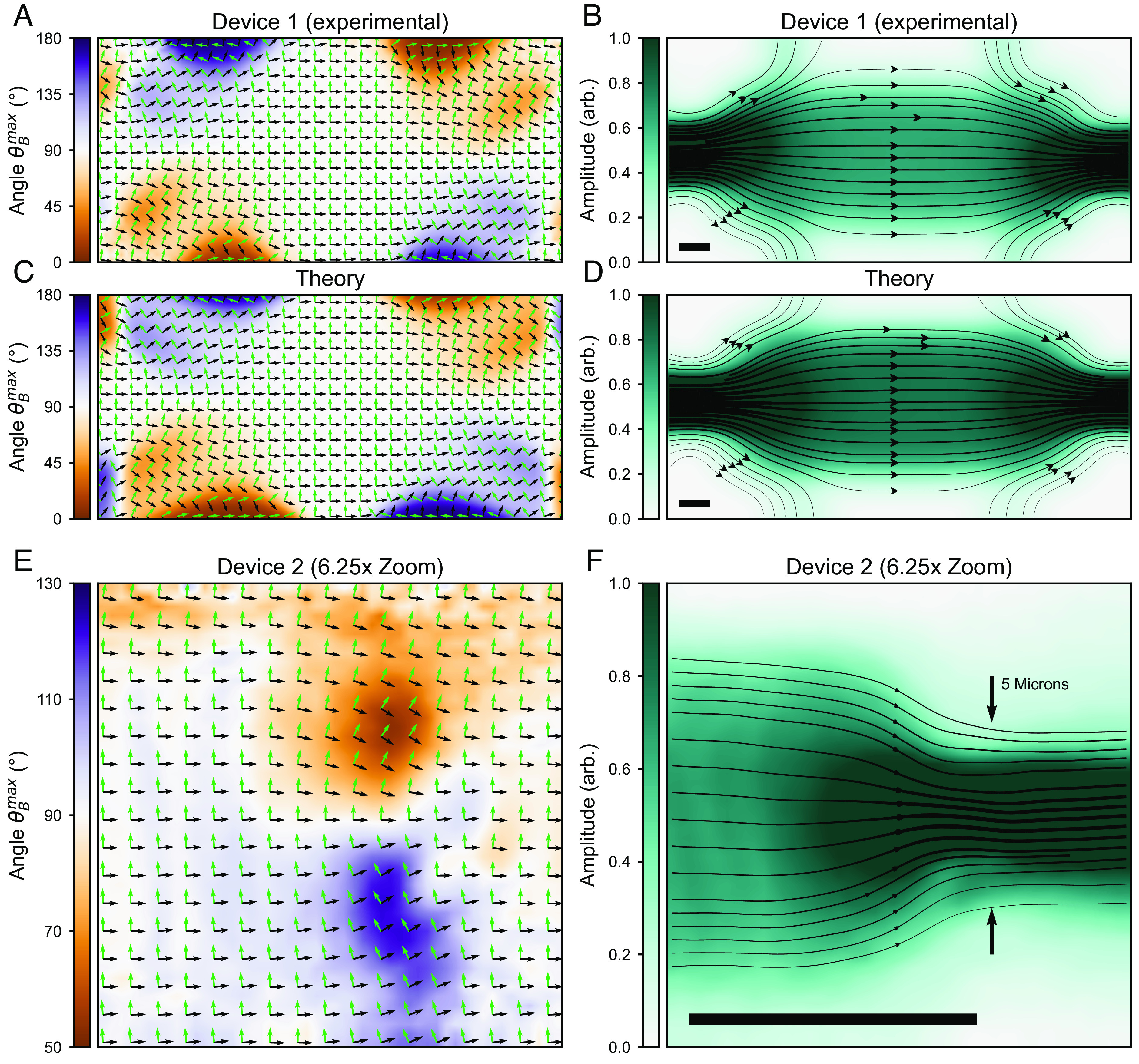
Imaging photocurrent streamlines through a micromagnetic heterostructure. (*A*) Experimental image of $\theta_{B}^{\max}$ for Device 1 (250 × 100 µm^2^) overlain with 2 vector fields: Green arrows indicate the direction of $\theta_{B}^{\max}$ at all points in space while black arrows indicate local photocurrent density. $\theta_{B}^{\max}$ determines the direction of the magnetic field that yields maximum detectable photovoltage signal and is always 90° off set from the photocurrent density. (*B*) Continuous flow-field interpolation of the photocurrent density vector field overlaying the sinusoidal fit amplitude $V_{m}$ at all points in space. (Scale bar, 50 μ.) (*C*) Theoretical image of $\theta_{B}^{\max}$ extracted from a Gaussian convolution of a theoretical calculation of $\vec{S}\left(\vec{r}\right)$ (*SI Appendix*, section S4.5). (*D*) Continuous flow-field interpolation of the photocurrent density field overlaying the theoretical fit amplitude $V_{m}$. (*E*) Experimental image of $\theta_{B}^{\max}$ for the right half of Device 2, (20 × 40 µm^2^). (*F*) Continuous flow-field interpolation of the photocurrent density in Device 2.

This phase map underpins a rich spatial pattern of streamlines through the device. To see this, in Fig. 3*A*, we superimpose a two-dimensional vector field over the image of $\theta_{B}^{\max}$ (*x, y*). Green arrows indicate the direction of the *B*-field that yields maximum photovoltage amplitude. Crucially, since the *B*-field controls the direction of the local charge current (Eq. **2**), we plot black arrows that indicate the direction of $\vec{j_{c}}$ that yields the maximum global photovoltage measured. By interpolating the black local current density arrows in Fig. 3*A*, we obtain a flow field through all points in the device, shown in Fig. 3*B* (*SI Appendix*, section S4).

The streamlines observed in Fig. 3*B* meander smoothly within the Pt film conforming to the device boundaries. These flow lines indicate the direction of *local* (photoinduced) charge flow that maximizes the *global* photovoltage response measured at current-drawing contacts. As such, they naturally explain the photovoltage polarity changes observed at corners and near the additional side contacts in Fig. 2*B*: Local charge current that is aligned to the flow lines generates maximum positive photovoltage (red diamond Fig. 2*B*), while antialigned local charge current generates negative photovoltage (blue triangle Fig. 2*B*). Conversely, $V_{\mathrm{ph}}$ becomes zero when the local photocurrent is perpendicular to the photocurrent streamlines, consistent with Eq. **1**. To further illustrate this, we numerically calculate $\vec{S}\left(\vec{r}\right)$ for the device and perform a Gaussian convolution to mimic the finite beam spot size. Fig. 3 *C* and *D* show the resulting field in the same manner as the experimental data (*SI Appendix*, section S4.5). The experimental data exhibit remarkable quantitative agreement to the photocurrent streamlines expected from the Shockley–Ramo theorem (7). Importantly, when voltage reading leads are reconfigured (e.g., by switching voltage readout to other contact pads in our device), the experimentally obtained photocurrent streamlines $\vec{S}\left(\vec{r},\;\right)$ similarly contort to favor strong photocurrent streamlines that directly connect the reconfigured voltage readout pads in accordance with that expected from Shockley-Ramo (see experimental $\vec{S}\left(\vec{r},\;\right)$ lines and theoretical comparisons in *SI Appendix*, section S4.6). Such examples vividly demonstrate the ability of our technique to sense different readout configurations as well as the concomitant direction of the preferred current flow.

These photocurrent streamlines are compressed within the Pt film as the boundaries become more restrictive. As shown in Fig. 3*B*, the photocurrent streamlines are highly sensitive to the device geometry: Photocurrent streamlines are uniform and dilute far from the boundaries yet converge and stream into a high-density bundle near narrow constrictions. To further illustrate this effect, we repeated the same measurements on a significantly smaller device using a nearly diffraction-limited beam spot to allow higher resolution measurements (*SI Appendix*, sections S2.1 and S4). With a device less than one-sixth the original size, Fig. 3*F* shows the same pattern of current compression near the contact as in Fig. 3*B*. For a finite spot-size laser illumination, the density of photocurrent streamlines corresponds directly to the intensity of the photovoltage response. As shown in Fig. 3 *B* and *F*, $V_{\mathrm{ph}}$ is strongly enhanced in regions of high photocurrent streamline density (e.g., along the narrow horizontal contacts) and suppressed in regions of low density.

**Fig. 4.**
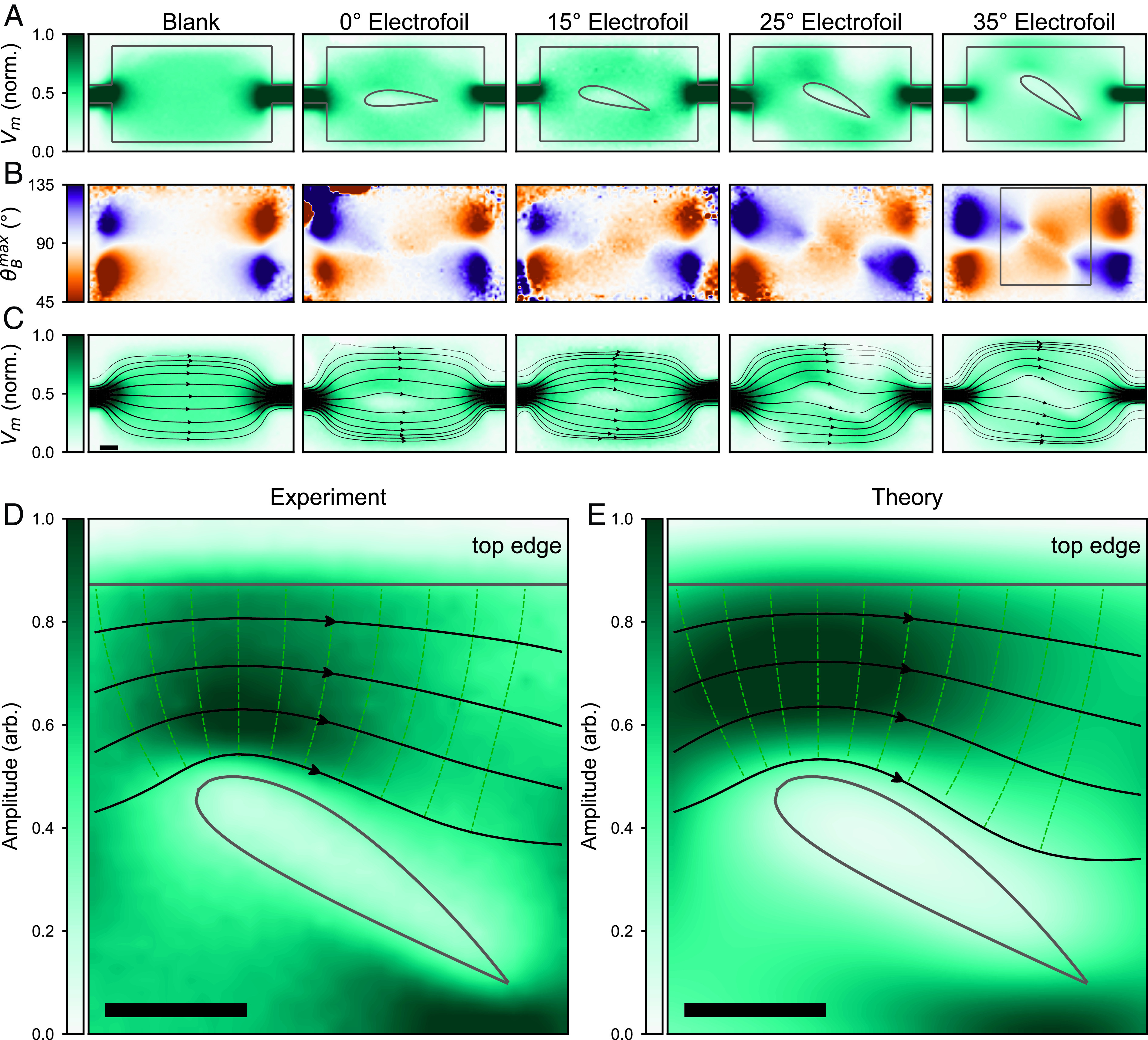
Contortion, compression, and expansion of photocurrent streamlines in a micromagnetic wind tunnel. (*A*) Images of the sinusoidal fit amplitude $V_{m}$ at all points in space for 5 different magnetic heterostructure devices. The blank device (far left) is a two-terminal rectangular plane (500 µm × 250 µm^2^). Electrofoil devices are two-terminal planes from which Pt is removed in the shape of aerodynamic airfoils with increasing angles of attack (labeled above), as outlined with solid lines. (*B*) Images of the angular phase shift of the fit $\theta_{B}^{\max}$ relative to $\theta_{B}$ = 0° at all points in space for each of the electrofoil devices. (*C*) Continuous flow-field interpolation of the photocurrent density vector field in *B* overlaying the sinusoidal fit amplitude $V_{m}$ at all points in space. Interpolated flow lines are weighted by the amplitude $V_{m}$. (*D*) Zoomed-in photocurrent streamline image and $V_{m}\left(x,y\right)$ amplitude for the 35° electrofoil device. (*E*) Theoretical simulation of $\vec{S}\left(\vec{r},\;\right)$ for the 35° electrofoil device. Solid black lines indicate current flow. (Scale bar, 50 μ.)

In addition to the dramatic current compression examined in Fig. 3, photocurrent streamlines—and the global photoresponse—can be manipulated far away from device boundaries in much the same way as fluid streamlines are guided to flow around an airplane wing. To test this, we repeated our experiment on specially fabricated devices with cross-sectional wing-shaped cut-outs, or electrofoils. Apart from an unpatterned blank device that is used as a control (Fig. 4 *A*, *Left*), the devices were created by removing regions of the Pt film in the shape of aerodynamic Clark Y airfoils (27) (Fig. 4); the electrofoils exhibit a convex upper profile and flat lower surface (gray outlines in Fig. 4*A*), and each is fabricated with an increasing angle of attack. Similar to the Hall bar device, we measure $V_{\mathrm{ph}}$ vs. *θ*_*B*_ and fit the resulting sinusoidal characteristics to obtain $V_{m}$ (*x, y*) and $\theta_{B}^{\max}$ (*x, y*) (Fig. 4 *A* and *B*). From these maps, we then produce images of the photocurrent streamline flow through the electrofoil devices (Fig. 4*C*) using the protocol described above.

Fig. 4*D* compares a detailed image of the photocurrent streamline flowing over the top surface of the 35° electrofoil to the theoretical expectations (Fig. 4*E*). At such a high angle of attack, photocurrent streamlines curve sharply to traverse the electrofoil (black lines Fig. 4*D*), creating regions of high- and low-density current flow. The photovoltage amplitude $V_{m}$ in Fig. 4*D* reaches a peak value above the leading (*Left*) edge of the electrofoil. By introducing the asymmetric cambered boundary, photocurrent streamlines are forced to curve more sharply around the leading edge of the electrofoil compared to the trailing edge. In this way, we gradually manipulate the photocurrent streamline density—and thus photovoltage intensity—along the length of the electrofoil surface.

Imaging the photocurrent streamline field $\vec{S}\left(\vec{r},\;\right)$ gives a unique fingerprint for each device, clearly identifying the lines along which photocurrent naturally flows. We emphasize that these photocurrent streamline patterns—while device specific—are agnostic to the mechanism through which spatially local photocurrents are generated. The experimental data of Fig. 4*D* directly visualize these natural photocurrent streamlines inside the electrofoil devices.

Even as $\vec{S}\left(\vec{r}\right)$, strictly speaking, only visualizes the directions along which spatially local photocurrent produces maximal photovoltage at remote contacts (an optoelectronic device property), it is nevertheless interesting to consider how it relates to other device characteristics. For instance, in the absence of an in-plane Hall effect, $\vec{S}\left(\vec{r}\right)$ can mimic the electric field spatially distribution of the device when it is operated purely electrically (7) (i.e., when only electric current is flowed from one contact to another without photoexcitation). Since $\vec{S}\left(\vec{r},\;\right)$ can be contorted and compressed (as shown in Figs. 3 and 4) close to sharp corners and near constricted geometries, we anticipate that the electric field distributions (when operated electrically) close to these regions are similarly morphed. This is especially important since the spatial distribution of electric fields, especially in nonuniform systems, can dictate the presence of nonuniform joule heating that affect device performance, e.g., for spintronics in metal/magnetic material systems (28, 29).

With imaging resolution spanning over an order of magnitude in spatial scale, we have shown that photocurrent streamline microscopy is a robust experimental tool for obtaining detailed visualization of photocurrent in quantum materials. The key requirements for this tool are a spatially tight laser illumination as well as directional control of *local* photocurrent (in our case, through *B*-field manipulation); when combined, this enables detailed visualization of *global* photocurrent streamline fields. While we have used a photoinduced Nernst effect in a micromagnetic device to enable directional control of the local photocurrent, other means of directional control of local photocurrent can be employed as well. For example, the bulk photovoltaic effect (1, 8) features local laser-induced photocurrent that has a direction that is sensitive to the polarization and helicity of light (30). Combined with a tight laser spot size, we anticipate that such light polarization control of the local photocurrent direction can also allow the ability to trace out the photocurrent streamlines in a wide variety of material systems that support the bulk photovoltaic effect (8); the key material requirement is the breaking of bulk inversion symmetry. Another exciting future prospect is the ability to exploit the sensitivity of $\vec{S}\left(\vec{r}\right)$ to the details of the conductivity tensor (7). For instance, irradiation incident on a sharp tip can produce near-field and highly localized photoexcitation (31) with extremely spatially tight local photocurrent generated; this may enable to probe nonlocal quantum geometric corrections to the conductivity (32–34) are difficult to access with conventional transport methods.

## Materials and Methods

The devices used in this work were designed using highly characterized micromagnetic heterostructures. All measurements were performed at room temperature using SMPM and analyzed using scalable methods of multidimensional data analysis. SMPM images are collected by a home-built data acquisition system and are stored and analyzed using a series of python scripts. We have measured several devices ranging in size from 40-μ wide to 500-μ wide using a photovoltage microscope with resolution ranging from 5 to 55 μ, thereby demonstrating the validity of the technique across an order of magnitude in spatial scale. The extended supporting information contains a detailed description of device fabrication, materials characterization, SMPM, imaging and analysis, thermo-spintronic response, and the experimental procedure. The extended supporting information also contains additional data and analysis regarding the isolation of the thermoelectric response, extraction of the magnetic-field dependent effects, and a detailed comparison between experiment and theory.

## Supplementary Material

Appendix 01 (PDF)

## Data Availability

All raw data and material analysis code (python) are available and material production is outlined in *SI Appendix*, *Supplementary Text*. Data (txt files) have been deposited in GitHub (https://github.com/qmolabucr/electrofoil) (35).
