# Supplementary material for: Mapping the intrinsic photocurrent streamlines through micromagnetic heterostructure devices
Source: Proc Natl Acad Sci U S A. 2023 Sep 18;120(39):e2221815120. doi: 10.1073/pnas.2221815120 (PMC10523491; doi:10.1073/pnas.2221815120)
Supplement: Supplementary file 1 — Appendix 01 (PDF) [file pnas.2221815120.sapp.pdf]

Supplementary Materials for

Mapping the intrinsic photocurrent streamlines through micromagnetic heterostructure devices

Authors: David Mayes[1,2,†], Farima Farahmand[1,2,†], Maxwell Grossnickle[1,2,†], Mark Lohmann[1], Mohammed Aldosary[1], Junxue Li[1], Vivek Aji[1], Jing Shi[1], Justin C.W. Song[3], Nathaniel M. Gabor[1,2*]

Affiliations:

[1]Department of Physics and Astronomy, University of California, 900 University Avenue, Riverside, California 92521, United States.

[2]Laboratory of Quantum Materials Optoelectronics, Materials Science and Engineering Building, Room 179, University of California, Riverside, California 92521, United States.

[3]School of Physical and Mathematical Sciences Division of Physics and Applied Physics, Nanyang Technological University, Singapore 637371.

*Correspondence to: nathaniel.gabor@ucr.edu.

† These authors contributed equally to this work

This PDF file includes:

Supplementary Text
Supplementary References **35-58**
Figs. S1 to S15

Supplementary Text

S1. Device Fabrication and Materials Characterization

The devices used in this work were designed using highly characterized micromagnetic heterostructures. As described below, detailed magnetic characterization of these metal/magnetic insulator devices has previously been reported in several manuscripts by our group and others. Our samples consist of thin platinum films (5 nm) patterned on a thin film of yttrium iron garnet (YIG) on top of gallium gadolinium garnet (GGG). The 80 nm YIG films are grown via pulsed laser deposition on GGG substrates with (110) crystal orientation. The details of the growth for the YIG thin films are described in an earlier report[36]. After confirming the atomic flatness and room temperature magnetic properties of the films we used e-beam lithography with PMMA resist topped with Elektra 92 (SX AR-PC 5000/90.2) conductive resist to create the different device patterns on the thin film surface. The pattern used for the electrofoil was designed using designCAD 2000 to mimic the Clark Y airfoil design. Each was rotated at different angles relative to the 0° (horizontal) design. After developing the patterns, we deposited the film in a sputtering chamber with base pressure of 5×10^{-8} Torr for deposition of 5nm Pt. This was then followed by liftoff of the negative exposure features in acetone. Optical images for several of the resulting device geometries can be seen in figure S1.

The coercive magnetic field of thin YIG samples has been found to be very small in both the in-plane[35] and out-of-plane[37] directions, measuring less than 0.2 T in all cases for films as thin as 4 nm and as thick as 35 nm[38]. External magnetic fields that exceed the coercive field saturate the magnetization of the YIG layer. The uniform B = 520 mT magnetic field in our measurement was chosen to sufficiently overcome the coercive field, thus ensuring that the magnetization - and therefore the spin polarization vector of any out-of-plane spin currents in the system - is aligned to the external B-field.

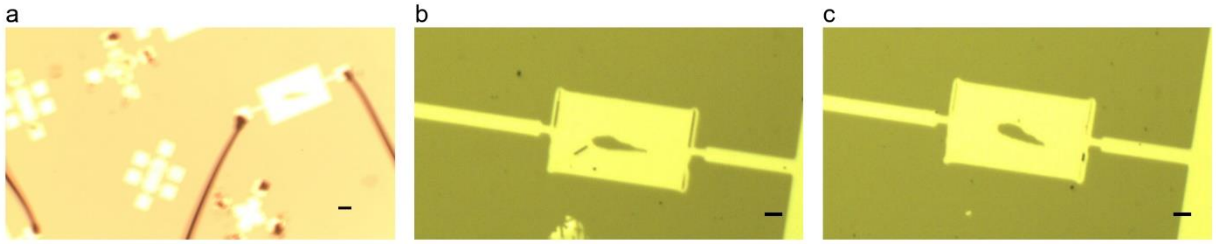

Figure S1.

Optical images for various device geometries. Optical images of the YIG substrate with several platinum devices are shown. Scale bars are each 50 microns long. ***a***, several closely packed platinum devices share the same YIG substrate, including two Hal bar, two terraced Hal crosses, and two electrofoil devices, with the 15° electrofoil device shown after wire bonding its two leads. The body of the Hal bar devices are 250x100 μm^2 while the electrofoil devices are each 500x300 μm^2. ***b***, expanded image of the 0° electrofoil device. ***c***, expanded image of the body of the 15° electrofoil device.

S2. Scanning Magneto-photovoltage Microscopy

All measurements were performed at room temperature using scanning magneto-photovoltage microscopy (SMPM) and analyzed using scalable methods of multi-dimensional data analysis developed in our previous work[39-40]. Section S2.1, presents a detailed description of the microscopy setup and its application while Section S2.2 presents details on the acquisition and analysis of the resulting imaging data.

S2.1. Scanning Magneto-Photovoltage Microscopy

Building on several previous thermomagnetic microscopes[20-25], SMPM is an experimental tool that utilizes both a strong, fully orientable magnetic field and a scanning laser to probe the thermoelectric response in novel micromagnetic materials. In this work, a scanning laser is used to generate a temperature gradient and induce the Longitudinal Spin Seebeck Effect (LSSE, discussed in detail in Section S3) over a small region of a micromagnetic device while measuring the potential difference across the device and exploring a broad parameter space. SMPM is automated to adjust power (via a neutral density filter), the 2D beam spot position (via a galvanometer), beam polarization, in-plane (x-y plane) magnetic field orientation, and the out-of-plane magnetic field orientation (x-z plane).

SMPM utilizes a high frequency mode-locked pulse laser to generate a photovoltage response, a rotatable Halbach array to produce a magnetic field, a lock-in amplifier, and home-built DAQ setup[40] to record the photovoltage response. The 830 nm wavelength, mode-locked, titanium-sapphire pulse laser (Coherent Mira Optima 900-F), with 200 fs pulses and a 76 MHz repetition rate is used to produce a spatially localized thermal gradient, which gives rise to a directional net spin-current density due to the LSSE. A set of two galvo-controlled mirrors are used to position and scan the beam while a set of optics and focus the beam on the back of either a microscope objective or a GRIN lens, resulting in a focused, scanning beam-spot. In addition to recording the photoresponse, a back-reflection amplitude is recorded for each measurement by introducing a 50/50 beam-splitter and photodiode to the beam path ahead of the galvo-controlled mirrors. A lock-in amplifier measures the relative voltage between two terminals and is synchronized to an optical chopper at low frequency of 4 Hz while the measurement time per point is 3.18 seconds.

Micromagnetic devices are mounted to a small PCB mount within the highly uniform B-field at the center of the Halbach array. The array, made from 6 N52 neodymium magnets precisely arranged to control the magnetic field (see Fig. S2a), measures B = 0.52 T at the sample position (see Fig. S2b). To allow for 90º out-of-plane rotation and 360º in-plane rotation, the array is attached to a rotation mount (Thor Labs stepper motor rotation mount), which in turn is mounted to a rotation stage (Thor Labs heavy duty rotation stage with stepper motor). As shown in Fig. S2b, measurements of the magnetic field within the active region of the Halbach array is highly uniform over an area of about 9 cm^2 in the center.

Orientation tests were carried out on an asymmetric device large enough to be photographed to ensure correct analysis of the multi-dimensional data. During this process, the device orientation was unambiguously established and magnetic field orientation relative to the device was determined. This crucial process allows verification of the signs of the fields and currents in the system and provides the precise definition of θ_B^{max} used throughout this manuscript.

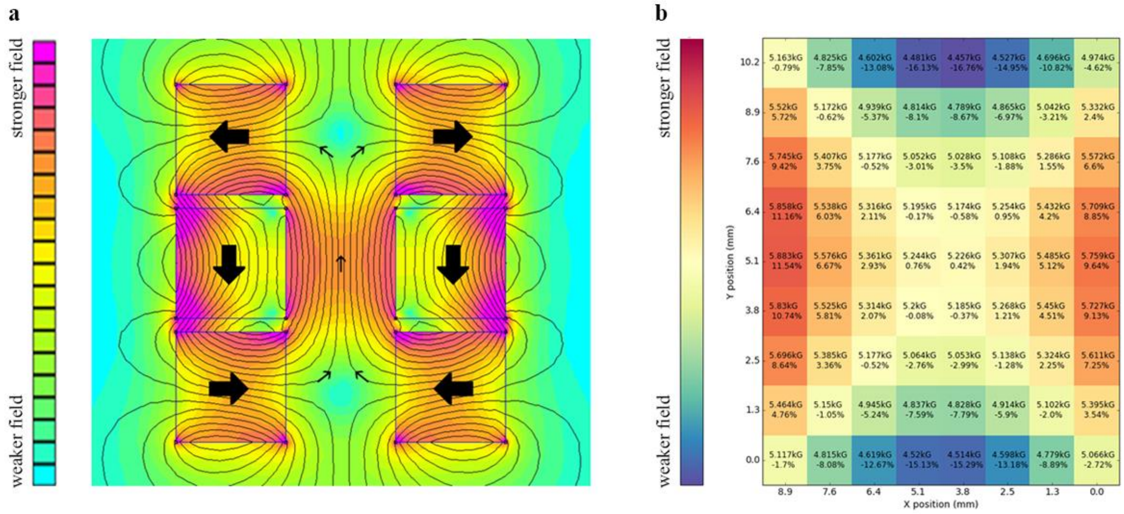

Figure S2.

Magnetic field strength vs. spatial position. ***a***, Field simulation of the Halbach array and the resulting magnetic field as illustrated by the magnetic field lines shown. Solid square lines represent outlines of N52 neodymium magnets. Arrows indicate the direction of the neodymium magnet's field. Curves are simulations generated by Finite Element Method Magnetics software. ***b***, Magnetometer readings of the in-plane magnetic field strength as a function of distance in the two-dimensional sample plane. In the center area, at the sample position, the field varies by less than 1%, while there is less than 20 % variance throughout the entire square-centimeter area pictured above.

S2.2. Imaging and Analysis

SMPM images are collected by a home-built data acquisition (DAQ) system and are stored and analyzed automatically using a series of python scripts introduced in a previous publication[40]. All automation tools are home-written using Python and are available upon request. As only the spatial location of the beam and in-plane magnetic field angle are used as parameters for the work presented here, the 3-dimensional datasets are easily visualized as a set of 2D images taken under different magnetic field orientations. The reflection amplitudes are stored in a separate dataset and are used to calibrate the SMPM and account for drift over long periods.

A typical device measurement, which acquires ~10^5 individual photovoltage and reflection measurements, takes between 5 and 15 days to complete, depending on the range of parameters. To account for sample drifting in over this time, drift correction is performed in the same manner as described in previous work[40], the results for which can be seen in Fig. S3. Note that during this process a 2D gaussian filter is applied to each reflection image, integrating out small features that can be seen in Fig. S3A but not in Fig. S3b.

All imaging data and analysis scripts used here are contained in a single Jupyter notebook, which is available upon request. The scripts contain well documented python code used for drift correction, Seebeck Effect extraction, and figure production. The analysis software relies heavily on the Matplotlib, NumPy, SciPy, and FiPy libraries.

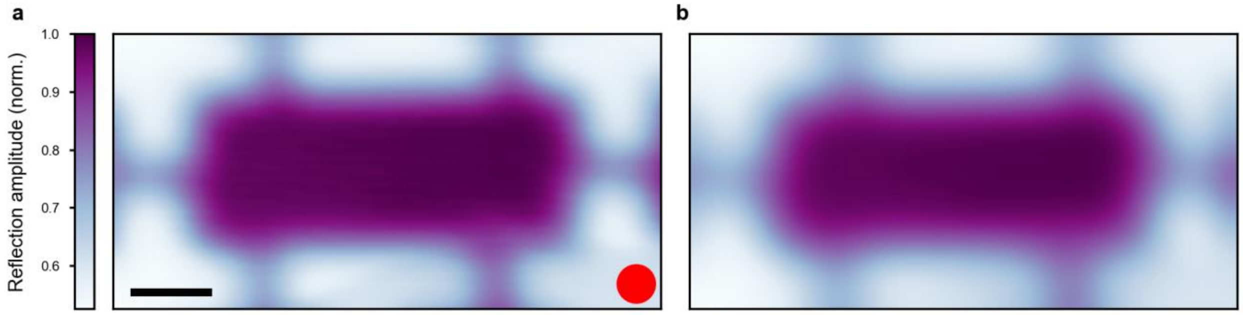

Figure S3.

Reflection images of the Hall bar device before and after drift-correction. ***a***, Back-reflection image of the Hall bar device prior to gaussian filtering and drift correction. ***b***, Back-reflection image after drift correction. Scale bar is 50 microns long while the beam spot (red circle) size is 27 microns FWHM. Small horizontal lines in ***a*** but not ***b*** are the result of 4Hz optical chopper interference.

S3. Thermoelectric and Thermospintronic Response in Micromagnetic Pt/YIG Devices

As discussed in section S4, there are both magnetic field-dependent and field-independent components to all datasets collected with SMPM. While the field-dependent photoresponse in Pt/YIG involves several effects, prior work[41-46] has demonstrated that, given the large out-of-plane temperature gradient, one effect dominates: The Longitudinal Spin Seebeck Effect (LSSE). While the field-independent, conventional Seebeck (thermoelectric) effect can be measured directly, the LSSE can only be detected via the Inverse Spin Hall Effect (ISHE), all described below.

S3.1. Conventional Thermoelectric (Seebeck) Effect

The conventional Seebeck effect, discovered by Alessandro Volta in 1794[47] while independently rediscovered by and named after Johann Seebeck in 1821, refers to the generation of a voltage across a conductor subject to a parallel temperature gradient, $V_{Se} = -S\vec{\nabla}T$. The underlying physical process driving this effect stems from the progressively higher kinetic energy imparted to charge carriers in hotter regions of a conductor, leading to a gradient in the mean free path of charge carriers and an overall outward flow. If there are metal junctions on either side of the heat source interrupting this electron flow, the difference between the Seebeck coefficients for the two metals, encoding the temperature dependence of each material's chemical potential, will cause a disproportionate charge build-up at the junction furthest from the heat source. While the Seebeck effect is described as an electric field, $E = -S * \nabla T$, across the device, it is important to note that the measurable voltage is the result of an electromotive force which seeks to restore equilibrium after the Seebeck effect disturbs local charge densities. This effect occurs independent of any magnetization or external magnetic field.

S3.2. Thermospintronic Response: The Longitudinal Spin Seebeck Effect (LSSE)

The Longitudinal Spin Seebeck Effect (LSSE) is the generation of a spin potential and an accompanying spin current in a magnetic material by means of an out-of-plane temperature gradient[41,46,48-50]. A conventional measurement of the LSSE requires one or more Peltier modules to control the temperature at the top and bottom of the device. This allows simple calculation of the temperature gradient across the magnetic material and will produce LSSE response without the Seebeck effect. Here we attempt to generalize these experiments by accounting for spatial variation in the effects.

While the mathematical form for j_S can be quite complicated, considering the density of spin carriers as well as the effective spin-mixing conductance at the interface between the magnetic and metallic layers, crystal symmetry dictates it is spatially independent, so it is expected that a consistent temperature gradient will produce the same spin current at any given point on a device[49]. It has been shown that for Pt/YIG, the spin current will flow anti-parallel to the temperature gradient[45,51]; that is, as the laser is used as a source for the temperature gradient, heating the platinum layer first, spin currents should experience a net flow from the platinum layer into the YIG.

In the classical Hall effect, a current running through a conductor interacts with a transverse magnetic field to produce a potential difference across the conductor which is orthogonal to both. The spin-current counterpart to this effect, the spin Hall effect (SHE), is a process in which an incoming charge current produces a spin current transverse to it. The SHE is observed in quantum heterostructure devices in which a normal metal with sufficiently high spin-orbit coupling (SOC) is bonded to either a ferromagnet or ferrimagnetic insulator (FMI), such as YIG. The underlying mechanism for the effect is the small torque experienced by charges propagating through the

normal metal which drives opposite spins in opposite directions[52]. The inverse spin Hall effect (ISHE) is the reverse process, in which a spin current traveling normal to the interface between a metal and FMI is polarized transverse to the spin current, resulting in a charge current orthogonal to both[45,52-54]. As a spin-current propagates out of plane it deflects charge carriers in the metal to conserve momentum. As charge-carriers of any spin are deflected in the same direction, depending only on magnetization and spin-current directions, an electric current is generated across the metal. This spin-to-charge conversion is described mathematically by $j_C = D_{ISHE}(j_S \times \sigma)$, where σ is the spin polarization vector of the material, j_S is the spin current from the LSSE described above, and $D_{ISHE} = \theta_{SH}\left(\frac{\hbar}{4\pi e}\right)$ is a material dependent constant. For a 15 nm platinum device at room temperature, θ_{SH} is very small and $D_{ISHE} \approx 1 \times 10^{-17}$Wb[55].

As platinum has strong SOC, the ISHE will convert a spin current traveling normal to its surface into a charge current orthogonal to both the spin current and the spin polarization vector. Given that in YIG the spin polarization vector can be saturated using a large in-plane magnetic field, applying a large out of plane temperature gradient to a Pt/YIG device in the presence of a saturating magnetic field in the plane of the device is expected to yield a charge current in the plane of the device orthogonal to the magnetic field. This process of generating a charge current from a thermally driven spin current is described generally by Equation 2 of the main text with a constant magnetic field and with the device-dependent Nernst-type coefficient derived from D_{ISHE} and j_S. For this experiment, the spin current points along the negative z axis (from Pt to YIG) and the magnetic field is held purely in-plane. The generated charge current is therefore also in-plane, with angle $\theta_B - \frac{\pi}{2}$. Thus, in the center of a large, symmetric device, it is expected that a maximum photoresponse would be measured when the external magnetic field angle is rotated to the angle $\theta_B^{max} = \frac{\pi}{2}$ where a maximum potential is measured when the current propagates in the positive *x*-direction.

S4. Experimental Procedure

In this section, the experimental procedure is discussed, including a careful walk-through of the data acquisition, data analysis, and image processing used in this manuscript. Section S4.1 describes the use of SMPM and our use of the analysis toolbox improved from those developed in our previous works[40]. While Section 4.1 provides details on the setup of the experiments, Sections S4.2 through S4.4 describe the signal analysis. Section S4.2 describes the handling of raw, drift-corrected datasets, including processing both reflection and photoresponse signals. Section S4.3 describes the process taken to extract the Seebeck Effect from the photoresponse dataset and subsequently separate the (magnetic field-*independent*) Seebeck and (magnetic field-*dependent*) Spin-Seebeck effects into separate datasets to be analyzed individually. This section also contains results from Seebeck measurements, which have been omitted from the main text. Finally, Section S4.4 discusses the analysis of the Spin-Seebeck (field-dependent) datasets, while the reader is encouraged to read Section S5 for fit accuracy details and elimination of additional effects.

S4.1. Data Acquisition

For this project we have measured several devices ranging in size from 40 microns wide to 500 microns wide using a microscope with resolution ranging from 5 to 55 microns, thereby demonstrating the validity of the technique across an order of magnitude in spatial scale. The focused Gaussian beam spot was measured using a knife-edge technique for all devices. For the initial Hal-bar device (device 1 in the main text, measuring 250 by 100 μm^2) as well as the terraced

Hall-cross (shown below), and un-patterned devices, we utilized a beam with a full-width-half-maximum (FWHM) of 23 microns. As the power was kept at 30 mW for all measurements, these devices were measured with the laser power density at $\sim 3.5 \times 10^3$ W/cm^2 yielding an out-of-plane thermal gradient at the Pt/YIG interface on the order of 1 K/µm. To measure the smaller device (20 x 40 µm^2, labeled as device 2 in the main text), the beam spot was reduced to the order of 5 microns with a power density of $\sim 8 \times 10^3$ W/cm^2. All electrofoil devices were measured with a much larger beam spot on the order of 55 microns, yielding a power density of $\sim 0.9 \times 10^3$ W/cm^2. This large beam spot is used to integrate the extremely weak LSSE signal in the linear response regime, confining our measurements to parameters in which photovoltage increases linearly with power. Voltage was measured across each device using a lock-in amplifier referencing an optical chopper. For device 2 in the main text, measured using a GRIN lens at high resolution, the transverse contacts were left floating. All other devices were measured using a microscope objective with grounded transverse contacts. Each measurement was taken with an optical chopper running at 4 Hz, an amplifier sensitivity of 10^{-7} volts, a time-constant of 300 ms, and a scan-rate of 0.374 pixels per second. The magnetic field was held purely in-plane for the duration of all measurements presented here.

S4.2. Raw Data and Preliminary Analysis

Once a measurement has been completed, and drift correction and other pre-analysis data-manipulation techniques have been performed as described in Sections S2 and S4.1, data analysis begins with analyzing the photovoltage datasets. The raw datasets are easily visualized as a series of 2D spatial maps of either the reflection or photoresponse amplitude resulting from illuminating a certain region of the device with the laser, given an evolving magnetic field orientation. The reflection and photovoltage datasets generated during measurement are imported into a Jupyter notebook for python-assisted analysis. As the magnetic field does not affect the reflection amplitude, the reflection datasets are averaged across the magnetic-field axis to produce a single image of the platinum layer of the devices. Fig. S4 shows the reflection amplitude for the Hall bar (Fig. S4a) and electrofoil devices introduced in the main text (Figs. S4 d-h) as well as 2 additional geometries studied here: an un-patterned Pt/YIG/GGG device (Fig. S4c) and a terraced Hall cross (Fig. S4b). The reflection images are used to guide the analysis of the photoresponse maps, providing an illustration of spatial displacement.

The first step in analyzing photovoltage datasets is to display them in image form. Fig. S5 is broken into four subfigures, each made of a series of raw photoresponse maps collected from various device geometries and varying by magnetic field-angle. The effects vary greatly depending upon the geometry, but when the field is parallel to the terminal axis, there is a consistently small photoresponse in the bulk of the device, while an orthogonal field produces a maximum (or minimum) response.

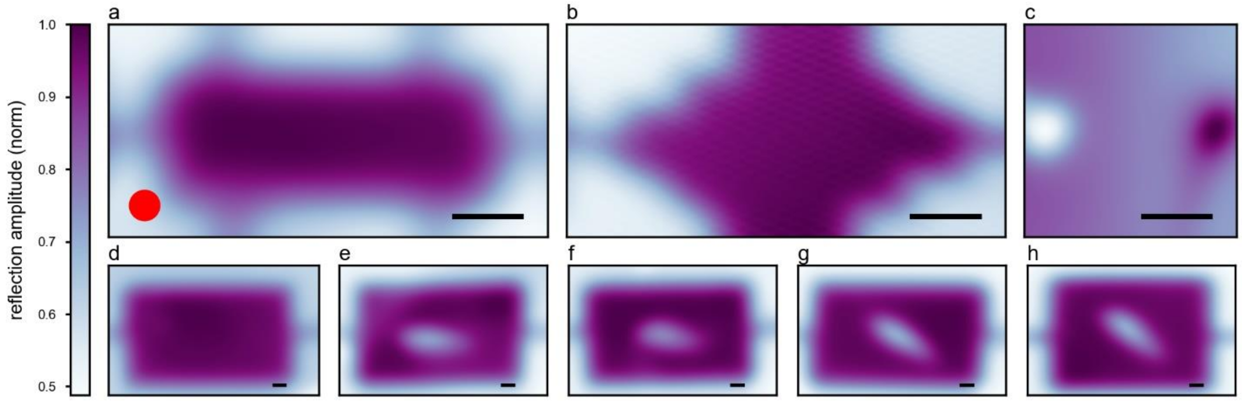

Figure S4.

Back-reflection amplitudes and optical images for various device geometries. ***a***-***h***, normalized reflection amplitude maps for various device geometries. Images shown are displayed after drift-correction (see Section S2 and Fig. S3). The reflection amplitudes illustrate the geometry of a device's platinum layer and are used for calibration and drift correction. ***a***, Hall bar device used throughout the main text. The beam spot is 27 microns FWHM. ***b***, Terraced Hall cross and ***c***, all platinum device, are introduced and referenced throughout this supplement. ***d***-***h***, The remaining electrofoil devices are used throughout the text and supplement. ***d***, Large 2-contact device. ***e***-***h***, Electrofoil devices arranged by increasing angle of attack: 0º, 15º, 25º, and 35º.

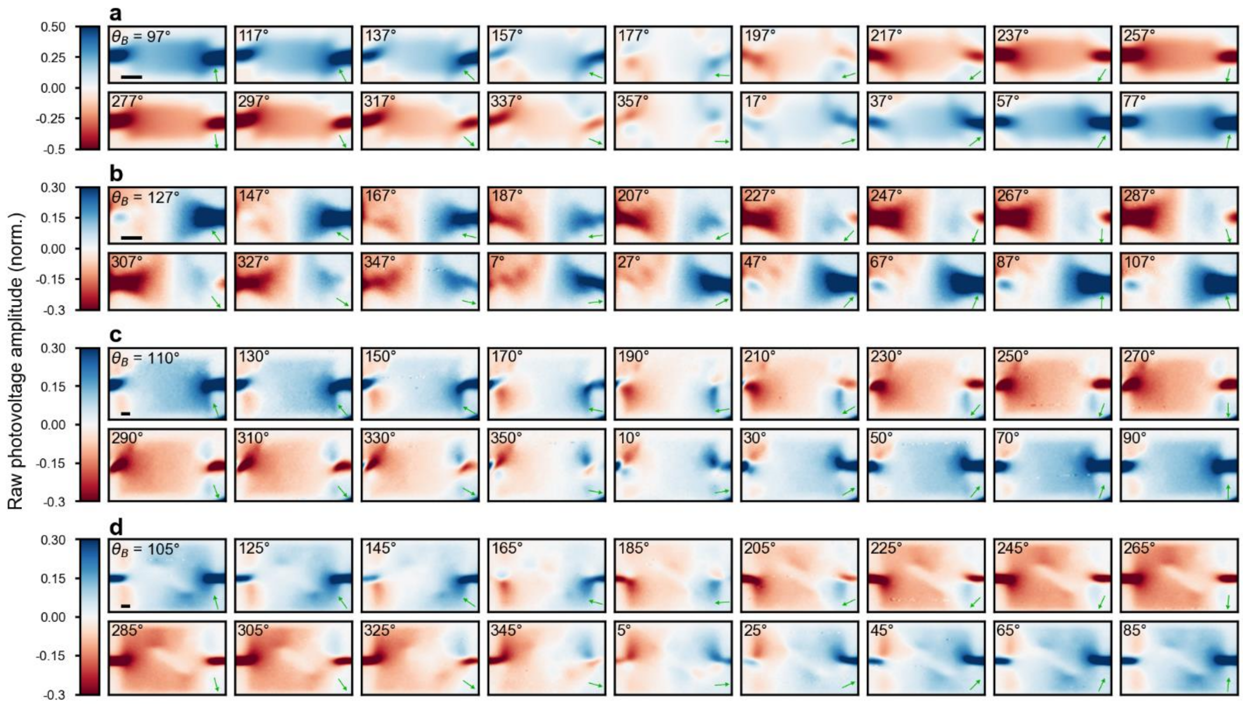

Figure S5.

Raw photovoltage dataset. Each subfigure contains 18 images, taken every 20º and represent the raw SMPM results. Four device geometries are depicted: ***a***, the Hall bar device used throughout the main text, ***b***, a terraced Hall cross, ***c***, the blank electrofoil device introduced in the main text, and ***d***, the 35º electrofoil device introduced in the main text. Magnetic field angle relative to terminal axis are indicated by θ_B in upper left corner of each image as well as green arrows in the bottom right corner. All images are normalized and saturated to highlight effects, as contact terminals generate a much larger signal than the bulk of the devices.

S4.3. Isolating the Thermoelectric (Seebeck) Effect

As the platinum devices are wire-bonded with aluminum wires, a Seebeck voltage is expected to be measured along with the desired ISHE response, proportional to the temperature differences between the contacts, implying that our signal will contain both magnetic field independent and dependent contributions. The raw photovoltage maps shown in Fig. S5 suggest that the field-dependent effects are sinusoidal in nature, thus the signal can be separated by taking the sum and difference of signals under opposite field orientation. This process is illustrated by Fig. S6, where the data in Figs. S5a and b are averaged together, point-by-point, to produce Fig. S6c, which in turn is subtracted from the data in Figs. S6a and b to produce Figs. S6d and e. These results are then subtracted from the raw datasets to produce the sinusoidally field-dependent datasets to be used throughout this text. As expected, the dashed line in Fig. S6f shows there is little variance in the Seebeck effect extracted data between different field orientations.

The field-independent Seebeck voltage is dependent only on the temperature gradient between the two aluminum wires on either side of the device, implying the signal will vary from positive to negative as the laser scans from one terminal to the other. As can be seen by comparing the various devices in Fig. S7, the effect is sensitive to anisotropy in each device but has consistent behavior overall. The data is otherwise as expected from the conventional thermoelectric response.

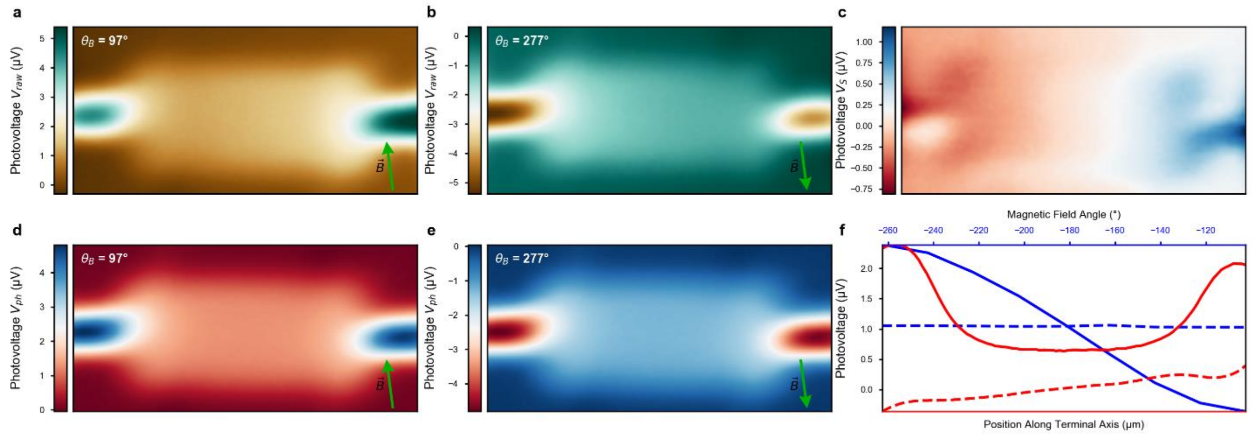

Figure S6.

Extracting the classic thermoelectric (Seebeck) Effect. Example illustration of the Seebeck extraction process using the same Hall bar sample as used throughout this work. Field orientation labeled by θ_B numbers in the upper left-hand corner and the directions of the green arrows. ***a***, Raw photovoltage amplitude map for the Hall-Bar device with a magnetic field orientation of 97°. ***b***, Raw photovoltage amplitude map for the Hall-Bar device with a magnetic field orientation of 277°. ***c***, A simple point-by-point average of the photovoltage amplitudes from 97° and 277° (the data in ***a*** & ***b***). ***d*** & ***e***, Subtracting the average (the data in ***c***) from the raw photovoltage data (the data in ***a*** & ***b***) produces V_{ph}. ***f***, Dashed lines represent a line trace of the Seebeck data while solid lines represent the spin-Seebeck data. The blue dashed line indicates the Seebeck effect is independent of the magnetic field.

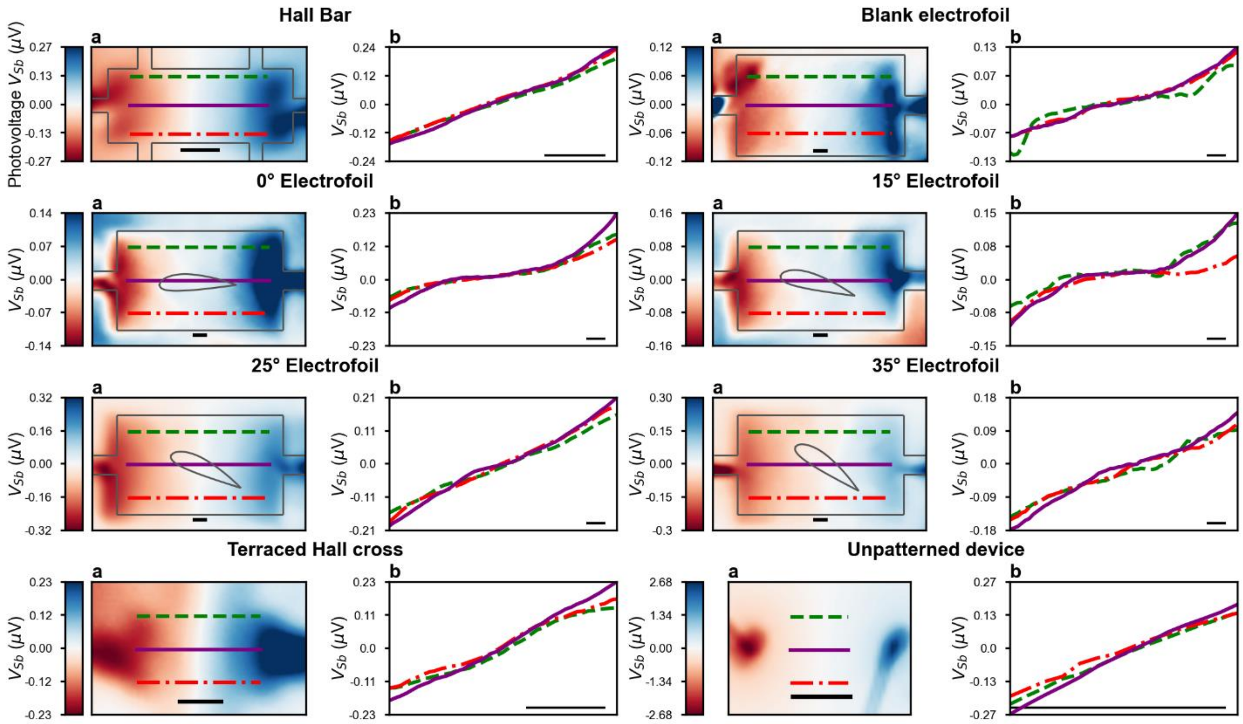

Figure S7.

The conventional thermoelectric (Seebeck) effect for various geometries. The Hall bar device is the same used throughout the main text, the electrofoil devices were introduced in the main text. The terraced Hall cross and the all-platinum device are introduced and referenced only in this supplement. ***a***, Photovoltage maps of the extracted Seebeck effect in various geometries. Solid black scale bars are 50 microns wide. Line traces are taken at three different positions along the devices and are represented by the colored solid and dashed used in both plots. ***b***, Spatial line traces of the photovoltage maps, depicting the consistency of the Seebeck effect across the devices. Colors and line styles represent line traces of the data in ***a***.

S4.4. Analyzing Magnetic Field-Dependent Effects

Fig. S8 shows the field-dependent dataset (V_{ph}) for different magnetic field angles for several different devices. Note the effect of the grounded contacts in the Hall bar device. The figure indicates that even when there are only probing contacts, there are still unusual edge effects when the magnetic field is parallel to the terminal axis. To examine this effect more thoroughly, each spatial point is fit to standard sinusoidal fitting function. After analyzing the results, the equation $V_{ph} = V_m \cdot cos(\theta_B - \theta_B^{max})$ is found to optimally fit the data, with all residuals to the fit lying well below the noise floor of our measurements. (Section S5) Here V_{m} is the maximum amplitude of V_{ph} and the cosine term captures the field-angle dependency. θ_B^{max}, for a given spatial position, is found to be the angle the magnetic field takes, referenced from the positive x-axis, which results in a maximum measurable electric field pointing in the negative x-direction. Figs. S9, S10, and S11, replicas of Fig. 2 in the main text, are shown here to complement Fig. 4 of the main text and highlight the consistency of the analysis across various geometries. The top panel in Figs. S9-S11 correspond to a selection of 10 out of 18 available magnetic field orientations that can be seen for most devices in Fig. S8.

Having reduced the 3-dimensional dataset for V_{ph} to two different 2-dimensional maps for V_m and θ_B^{max}, we begin to study the anomalous spatial dependence in more detail. As the underlying response is field-dependent, it is reasonable to assume that $\theta_B^{max}(x,y)$ and $V_m(x,y)$ can be treated as components of a vector field. As a first step in analyzing the discrete data this way, we plot $V_m(x,y)$ and overlay it with a field of arrows pointing in the direction $\theta_B^{max}(x,y)$. The arrows are generated automatically from the phase data using the python graphical plotting function quiver from the matplotlib library. Fig. 3a of the main text highlights the results of this method. We then use the python graphical interpolation function streamplot from the matplotlib library to interpolate between the arrows and produce a flow field through the device, shown in Fig. 3b and 4c of the main text. In a similar manner, we use the streamplot method to plot the interpolation of the field orthogonal to $\theta_B^{max}(x,y)$ at each point in space, *x* and *y*, a field explored by the main text and illustrated in Fig. 4d.

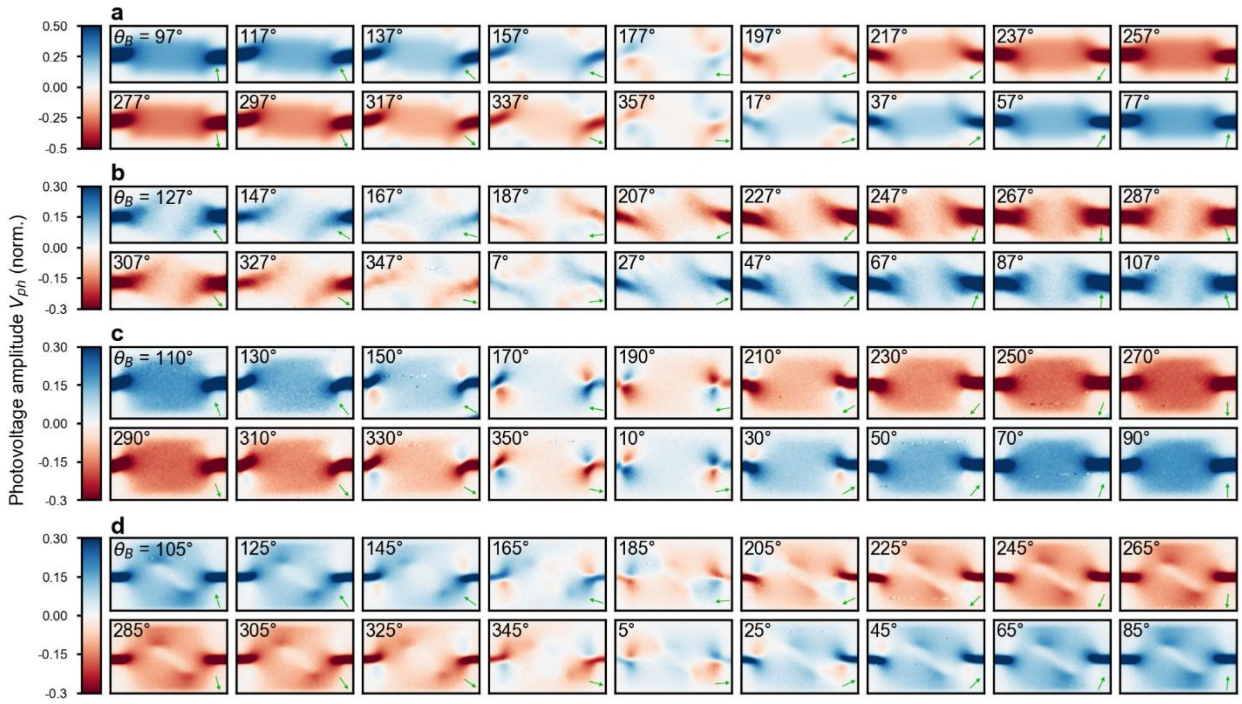

Figure S8.

Magnetic Field-dependent dataset. Contains the same devices and angles as used in Fig. S5, now with the Seebeck effect extracted from each plot, as illustrated by Fig. S6. Each subplot, differentiated by device geometry, contains 18 photovoltage images, taken every 20°. ***a***, the Hall bar device used throughout the main text. ***b***, The terraced Hall cross device. ***c***, The blank electrofoil device, and ***d***, the 35° electrofoil device. All images are normalized and saturated to highlight their effects. Magnetic field angle relative to terminal axis is indicated by θ_B in upper left corner of each image and by green arrows in the bottom right corner.

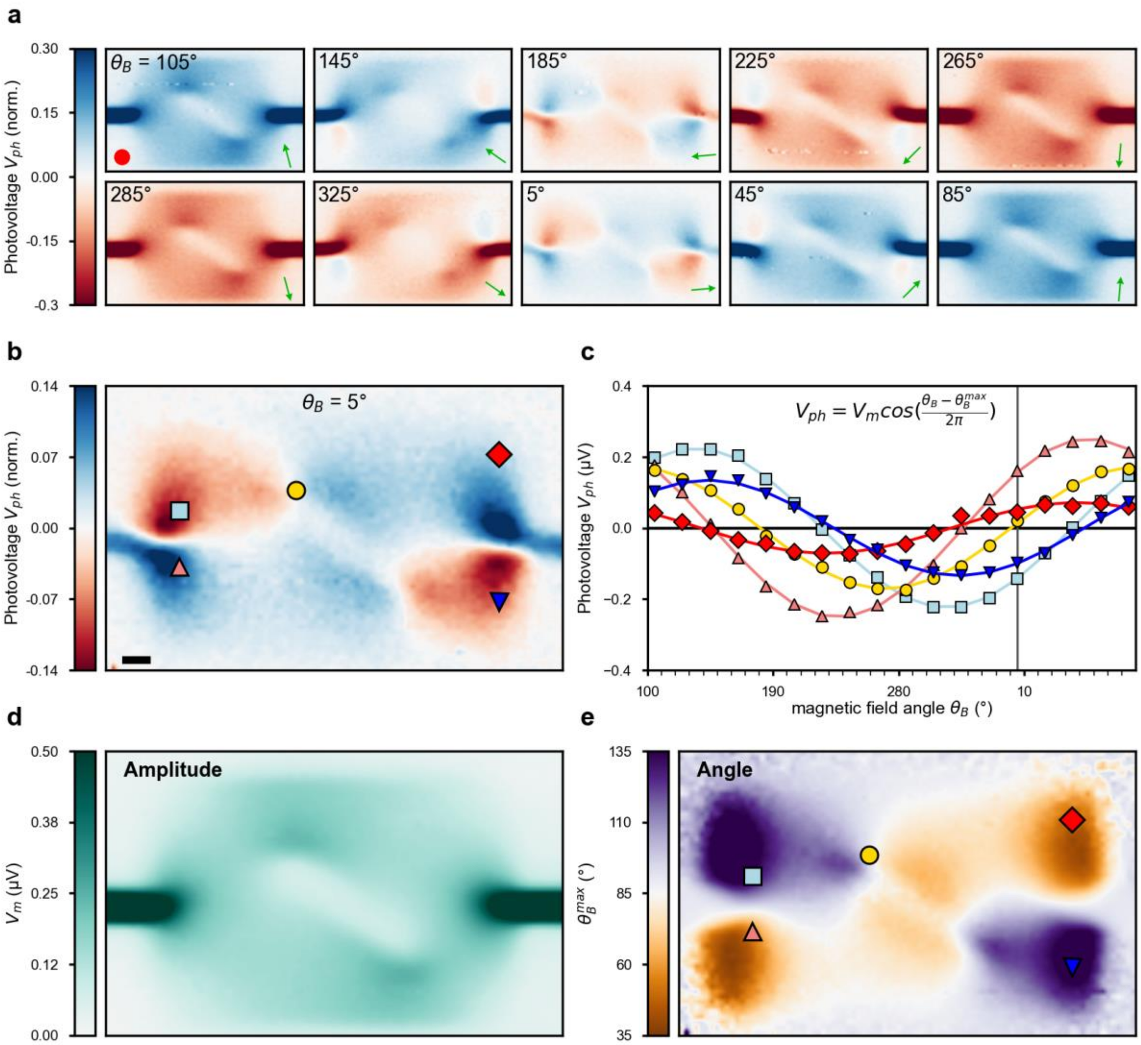

Figure S9.

Analyzing Field Dependent Effects in the 35º electrofoil device. Replica of Fig. 2 from the main text, using the 35º electrofoil device in place of the Hall bar device originally shown. ***a***, A subset of magnetic field-dependent photovoltage maps at 10 different magnetic field angles. θ_B is labelled top left corner, B-field direction is indicated by green arrows. Red circle indicates the FWHM of the beam spot (FWHM = 54 µm). ***b***, Detailed view of the anomalous photovoltage features at θ_B = 357º. Scale bar 50 microns for all images. ***c***, V_{ph} vs. θ_B for 5 points marked in ***b***. Data points share the same colors and shapes as in ***b***. At each point in space, V_{ph} vs. θ_B is fit to the function $V_{ph}(\theta_B) = V_m \cos(\theta_B - \theta_B^{max})$, shown as corresponding solid lines. ***d***, Image of the sinusoidal fit amplitude V_m at all points in space. ***e***, Image of the angular phase shift of the fit θ_B^{max} relative to θ_B = 0º at all points in space. Marked points are the same as those in ***b***, corresponding to the V_{ph} vs. θ_B data in ***c***.

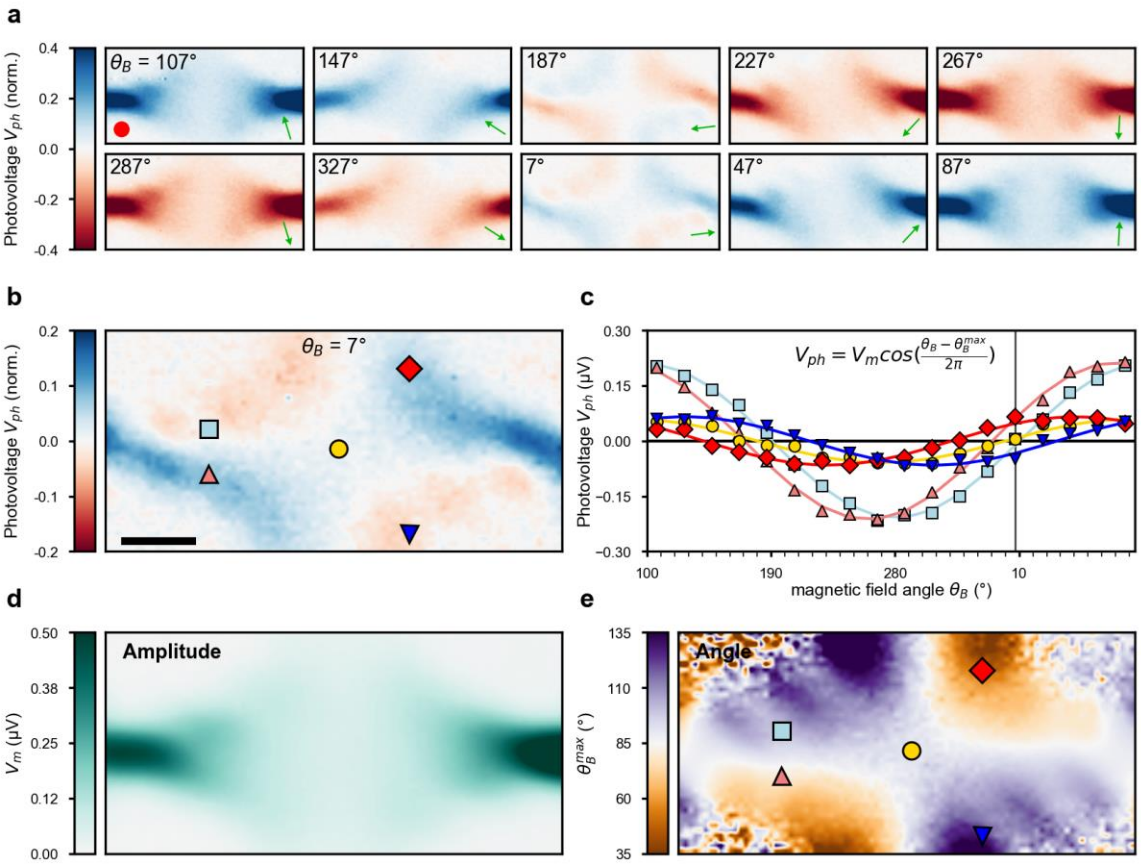

Figure S10.

Analyzing Field Dependent Effects in the Terraced Hall Cross device. Replica of Fig. 2 from the main text, using the terraced Hall cross Pt/YIG device in place of the Hall-bar device originally shown. ***a***, A subset of magnetic field-dependent photovoltage maps at 10 different magnetic field angles. θ_B is labelled top left corner, *B*-field direction is indicated by green arrows. Red circle indicates the FWHM of the beam spot (FWHM = 27 µm). ***b***, Detailed view of the anomalous photovoltage features at θ_B = 357°. Scale bar 50 microns for all images. ***c***, V_{ph} vs. θ_B for 5 points marked in ***b***. Data points share the same colors and shapes as in ***b***. At each point in space, V_{ph} vs. θ_B is fit to the function $V_{ph}(\theta_B) = V_m \cos(\theta_B - \theta_B^{max})$, shown as corresponding solid lines. ***d***, Image of the sinusoidal fit amplitude V_m at all points in space. ***e***, Image of the angular phase shift of the fit θ_B^{max} relative to θ_B = 0° at all points in space. Marked points are the same as those in ***b***, corresponding to the V_{ph} vs. θ_B data in ***c***.

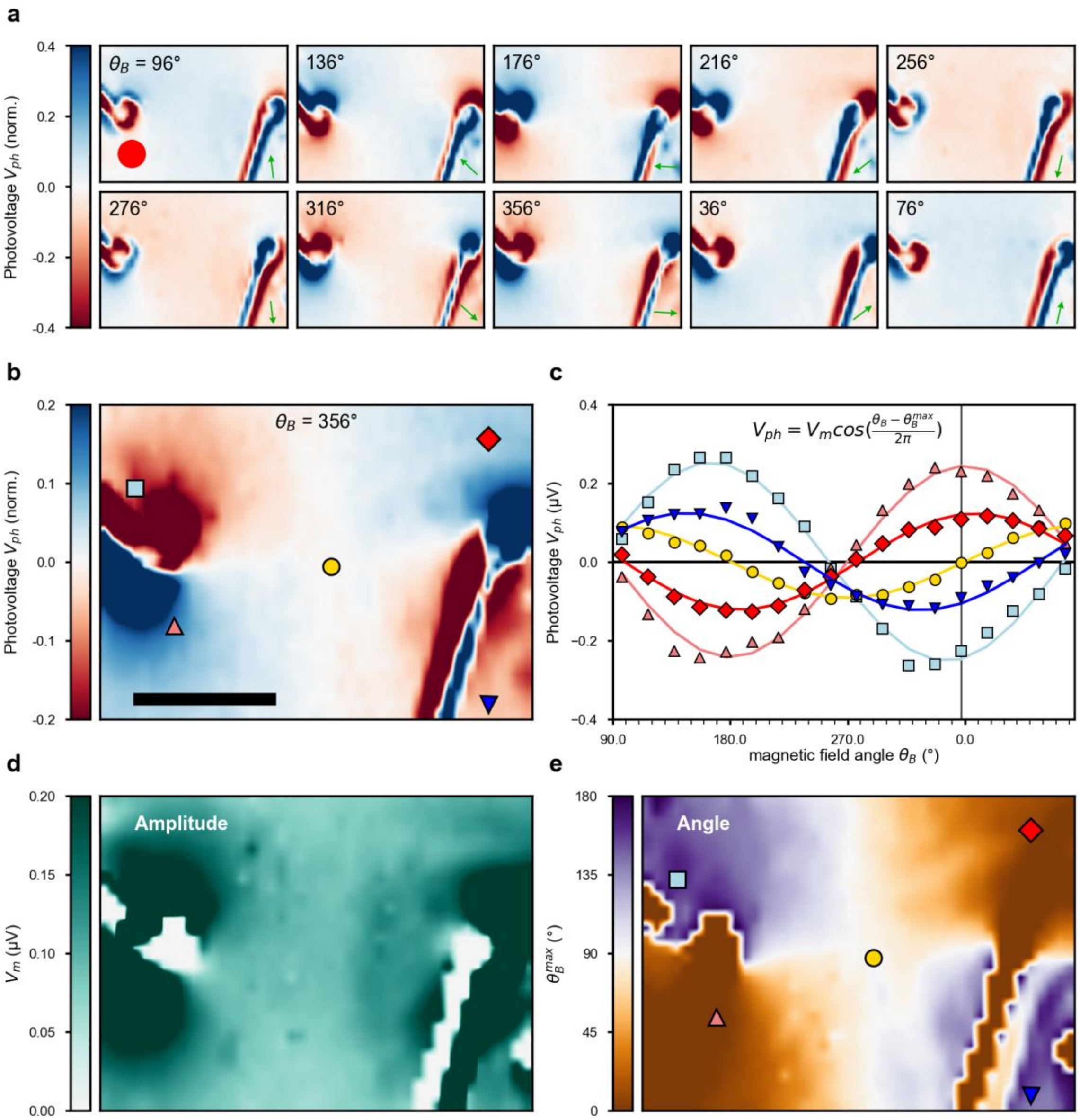

Figure S11.

Analyzing Field Dependent Effects in an un-patterned Pt/YIG Device. Replica of main text Fig. 2 using an un-patterned Pt/YIG device. ***a***, A subset of magnetic field-dependent photovoltage maps at 10 different magnetic field angles. θ_B is labelled top left corner and is indicated by green arrows. Red circle indicates the FWHM of the beam spot (FWHM = 27 μm). ***b***, Detailed view of the anomalous photovoltage features at θ_B = 357°. Scale bar 50 microns for all images. ***c***, V_{ph} vs. θ_B for 5 points marked in ***b***. Data points share the same colors and shapes as in ***b***. At each point in space, V_{ph} vs. θ_B is fit to the function $V_{ph}(\theta_B) = V_m \cos(\theta_B - \theta_B^{max})$, shown as corresponding solid lines. ***d***, Image of the sinusoidal fit amplitude V_m at all points in space. ***e***, Image of the angular phase shift of the fit θ_B^{max} relative to θ_B = 0° at all points in space. Marked points are the same as those in ***b***, corresponding to the V_{ph} vs. θ_B data in ***c***.

S4.5. Comparison to results predicted by S-R

According to the Shockley-Ramo theorem, a moving charge in a two-dimensional conductor of arbitrary geometry can produce a measurable electric signal only when the dot product of its velocity with the field S(r) is non-negligible. Thus, the field S(r) moderates the electric signal of a given device and is critical in understanding classical charge transport. As discussed in the main text, the field S(r) can be calculated by solving a Laplacian with appropriate boundary conditions. The python library FiPy[56] is used to theoretically model S(r) by solving for the Laplacian using the finite volume method. Boundary conditions are supplied to FiPy by means of a finite-element polygon mesh created in the application Gmsh[57]. Gmsh was used as a CAD engine for recreating the device geometry before generating the mesh file used in FiPy. The settings used by Gmsh as well as the resulting meshes are available as standalone files upon request.

To compare the theoretical field generated by FiPy with the experimental results, it is necessary to run the field through a convolution filter that will mimic the finite-size of the beam spot. FiPy's results, however, are not linearly spaced and consist of a set of spatial points along with the corresponding x and y directions of the vector field. Before running it through any convolution filters, the data is interpolated along a set grid that is slightly larger than the experimental images, allowing for the image to be cropped after the convolution filter is run. As can be seen in the main text (Fig. 3), the resulting theoretical fields show remarkable similarity to the experimental data in all devices tested.

S4.6. Alternative Wiring Configurations

While the typical setup for the standard six-contact Hal bar device used in this text measures the potential difference across the length of the device, measurements of several other wiring configurations can also be performed to compare against their expected behavior. Fig. S12 illustrates five different wiring configurations of the same Hal-bar device, where configuration three represents the typical setup. For each configuration, the figure depicts a wiring diagram, the field independent Seebeck response, and the field dependent response at two, opposite magnetic field angles for each of the wiring configurations. This is compared to several other configurations, including measuring between two contacts on the same side of the device in configuration two, around a corner in configuration four, and between the bridged contacts on the top and bottom of the device in configuration one. Each response is as would be expected, with large, equal and opposite response at each point in the regions current would be expected to flow if the contacts were biased appropriately.

To illustrate this further, device 2 of the main text uses a six-contact Hall bar design, just as device 1, however it is measured with floating side contacts. As can be seen in Fig 3 of the main text and in Fig. S13 below, the two devices show similar responses throughout the body of the devices and near the main contacts, with a relatively consistent amplitude throughout the body corresponding to a constant θ_B^{max} of 90° and with accentuated amplitude at the main contacts. There is, however, a significant difference in responses in the region of the side contacts. In Fig. 3b, the grounded device 1 shows a large response in the regions of each of the side contacts, while Fig. 1a shows that current in these regions must travel nearly perpendicular to the primary axis to be detected. In contrast, Fig. S13c shows that any response in the region of the side contacts of device 2 falls below the noise threshold.

To generate the theoretical models, both devices are given boundary conditions consisting of positive and negative unit bias supplied to the two main contacts, while device 1 has grounded side contacts and device 2 has floating side contacts. As demonstrated by Fig. 3c and 3d and discussed

in the main text, the theoretical model, and the experimental measurements for device 1 show remarkable similarity. The amplitude for device 2, while noisier than device 1, also shows strong similarities to the theoretical model, shown in Figs. S13c and S13d. The lack of amplitude in the regions of the side contacts, however, leads to large discrepancies between the experimental and theoretical maps of θ_B^{max} shown in Figs. S13a and S13b, respectively. While the theoretical model predicts a similar behavior in θ_B^{max} to device 1, the experimental measurements show only noise in those regions. The higher noise level may be due to a large beam spot size, on the order of 5 microns, compared to the device itself, 20 by 40 microns, as well as additional effects stemming from the profile of the beam itself.

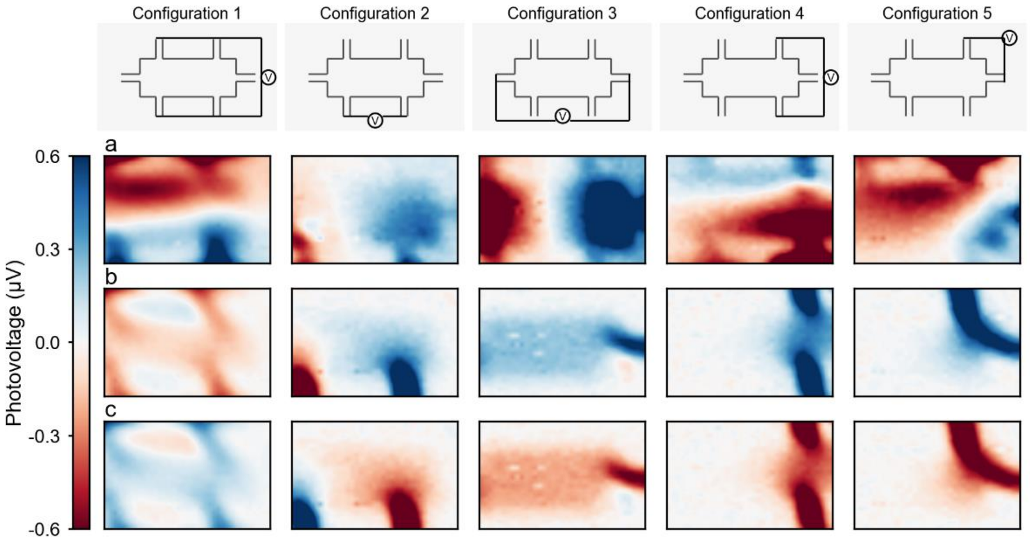

Figure S12.

Alternative Wiring Configurations. ***top***, wiring diagram for configuration. ***a***, extracted field-independent Seebeck Effect generated by each configuration. ***b,c***, the field-dependent SSE/ISHE data for two opposite magnetic field angles at approximately 45 and 225 degrees relative to the devices. ***Configuration one***, potential difference is measured across the device with the two side contacts on the top and bottom connected. ***Configuration two,*** potential difference is measured across the two "bottom" contacts. ***Configuration three,*** potential difference is measured across the device in the same manner as throughout the rest of this text. ***Configuration four,*** potential difference is measured across the two "right-hand side" contacts. ***Configuration five,*** potential difference is measured across a corner, between the right-hand side main contact and the top-right side contact.

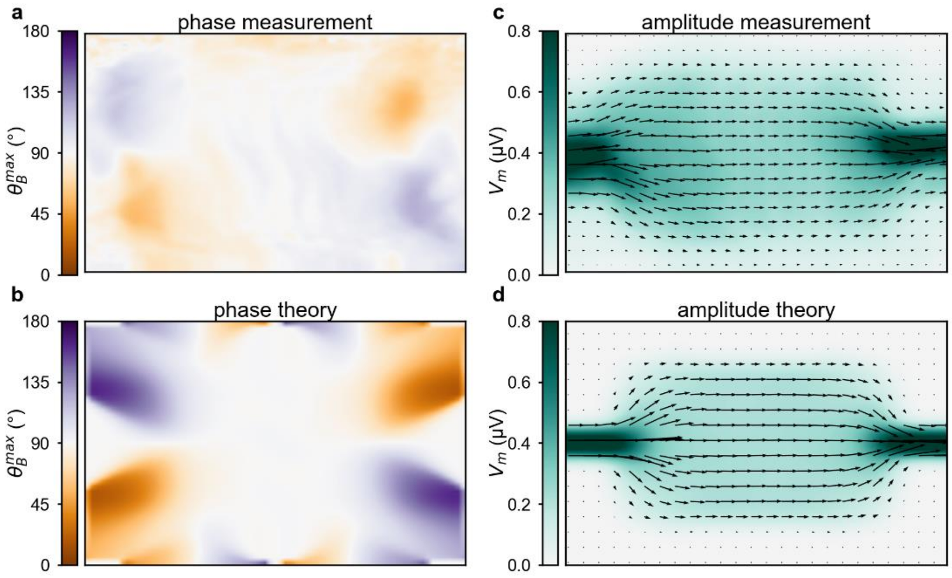

Figure S13.

Comparison of an Alternative Configuration with Theory. The results of measuring a Hal bar device with floating side contacts is compared against theoretical expectations. ***a,c***, results from fitting the data from experimental measurements of Device 2 a six-contact Hal bar device (40 x 20 microns) where all side contacts are left floating. ***b,d***, theoretical model of a six-contact Hal bar device setup with positive and negative unit potentials supplied to two side contacts on one end of the device while the other contacts are left floating. ***a,b***, experimental and theoretical amplitude maps for the device. ***c,d***, experimental and theoretical images of image of θ_B^{max} at each point overlain with a field of arrows pointing in the direction of current flow and weighted by the amplitude at that point.

S5. Discounting Competing Magnetic Effects

As the spatial dependence of V_{ph} was not predicted from the ISHE alone, it is reasonable to ask if other magnetic effects could be present in the system. As discussed in Section S4, the Seebeck effect data is calculated by averaging the photovoltage amplitudes for a single spatial point with opposite magnetic field signs – so the Seebeck effect data is still a dataset with a 3rd dimension that has been cut in half. As can be seen in Fig. S14, analyzing the standard deviation of amplitude for the Seebeck effect data across the different magnetic field orientations indicates that any additional effects (other than Seebeck effect or LSSE/ISHE) are negligible. Indeed, in this work, any additional field-dependent effects must lie well below the highly sensitive voltage noise floor of our measurement. As a control measurement, another Hall bar device was fabricated with only the platinum and GGG layers. As can be seen in Fig. S15, only the conventional Seebeck Effect is observed.

The Hall effect, discovered by Edwin Hall in 1879[58], is a result of the Lorentz force on moving charges in a magnetic field and is characterized by a current deflection in the direction orthogonal to both the current and the external magnetic field. As this experiment relies heavily on the use of a Halbach array which produces a large magnetic field to saturate the magnetization of the YIG, the influence of this effect on the experimental results must be accounted for. The Lorentz force, $\vec{F} = q\vec{v} \times \vec{B}$, exerts a force on the charge carriers causing them to drift. For the duration of this experiment, $\vec{B}$ is purely in-plane and voltage probes are located on opposite edges of the devices, so measurable current also occurs in-plane. Thus, the Hall effect should generate some unmeasurable out-of-plane current. However, the Hall effect should also be present in the same devices missing the YIG layer. As the Pt/GGG signal, shown in Fig. S15 contains only the Seebeck effect and all other effects can account for less than 0.1% of the total signal, the Hall effect should also be negligible in our system. This same measurement also discounts the Nernst effect, in which $V = B \times T$. The anomalous versions of these effects, which rely on magnetization (M) rather than B, must also be accounted for. In a similar system with 5nm-thick Pt layers, it is known that the anomalous and ordinary Hall effects should be of comparable magnitude[44]. As the Hall effect has been shown to be negligible in these systems, it is assumed that the anomalous Hall effect is negligible as well. In the literature, several experiments have failed to detect the anomalous or proximity Nernst effects in similar systems[32]. While this lack of findings does not definitively rule out the possibility of interference with these effects, it indicates they are likely very small if present at all.

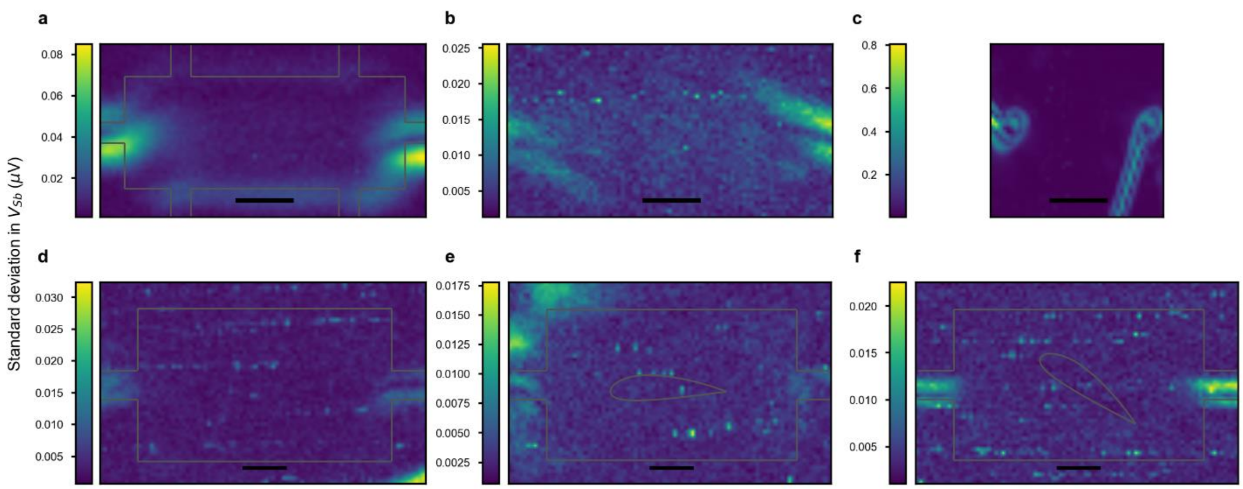

Figure S14.

Standard deviation in Seebeck extraction across scans taken at various magnetic field orientations. Plots are separated by device geometry, ***a***, is the standard Hall Bar device used throughout the text, ***b***, is the terraced Hall cross, ***c***, is the un-patterned Pt/YIG device, ***d***, is the large, 2-contact device, ***e***, is the 0° electrofoil and ***f***, is the 35° electrofoil. Scale bars are each 50 microns long. Each spatial position on each device is measured 18 times, once every 20°. The Seebeck effect is extracted by averaging two measurements with opposite field angles, resulting in 9 individual results for the averaging process; panels ***a-f*** represent the standard deviation of these averages.

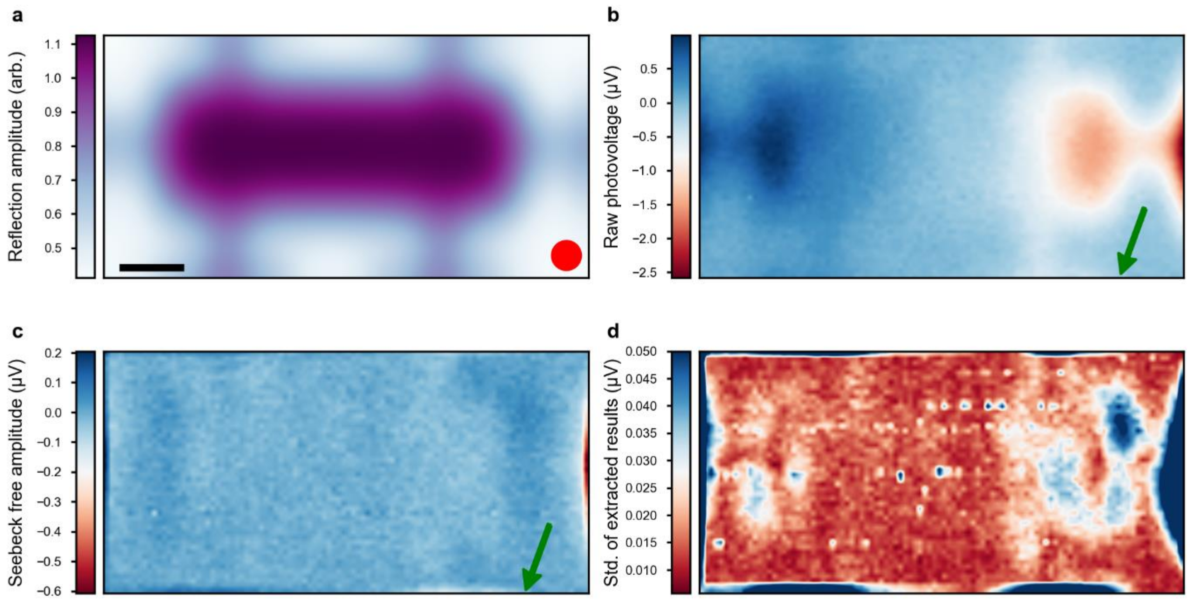

Figure S15.

Null Results of Pt-GGG measurement. ***a***, A reflection image of the Pt-GGG Hall bar device. The device geometry is the same as the Hall bar used throughout the text. The beam spot is 27 microns FWHM and the scale bar is 50 microns long. ***b***, Raw photovoltage response for the Pt-GGG device. Black arrow represents the magnetic field angle when the photovoltage was recorded. ***c***, Pt-GGG measurement after Seebeck results have been subtracted, measured at the same angle as ***b***. ***d***, Standard deviation of the Seebeck-subtracted signal across all measured angles, indicating the results in ***c*** are representative of all field orientations.

Supplementary References

35. Schreiber, F., et al. Magnetic sensitivity distribution of Hall devices in antiferromagnetic switching experiments. *Phys. Rev. Appl.* **16**, 064023 (2021).
36. Tang, C., et al. Exquisite growth control and magnetic properties of yttrium iron garnet thin films. *Applied Phyics Letters* **108**, 102403 (2016).
37. Castel, V., Vlietstra, N., van Wees, B. J., Youssef, J.B. Frequency and power dependence of spin-current emission by spin pumping in a thin-film YIG/Pt system. *Physics Review B* **86**, 134419 (2012).
38. Vlietstra, N., Shan, J., Castel, V., van Wees, B. J. Spin-Hall magnetoresistance in platinum on yttrium iron garnet: dependence on platinum thickness and in-plane/out-of-plane magnetization *Physics Review B* **87**, 184421 (2013).
39. Arp, T. B., Pleskot, D., Aji, V., Gabor, N. M. Electron–hole liquid in a van der Waals heterostructure photocell at room temperature. *Nature Photonics* **13**, 245–250 (2019).
40. Arp, T. B., Gabor, N. M. Multiple parameter dynamic photoresponse microscopy for data-intensive optoelectronic measurements of van der Waals heterostructures. *Review of Scientific Instruments* **90**, 023702 (2019).
41. Uchida, K., et al. Observation of longitudinal spin-Seebeck effect in magnetic insulators. *Appl. Phys. Lett.*, **97** 172505 (2010).
42. Wang, S., et al. Spin Seebeck effect and spin Hall magnetoresistance at high temperatures for a Pt/yttrium iron garnet hybrid structure. *Nanoscale* **7**, 17812-17819 (2015).
43. Miao, B. F., Huang, S. Y., Qu, D., and Chien, C. L. Absence of anomalous Nernst effect in spin Seebeck effect of Pt/YIG. *AIP Advances* **6**, 015018 (2016).
44. Meyer, S., R., et al. Anomalous Hall effect in YIG|Pt bilayers. *Applied Physics Letters* **106**, 132402 (2015).
45. Arana, M., et al. Spin to charge current conversion by the inverse spin Hall effect in the metallic antiferromagnet Mn2Au at room temperature. *Physics Review B* **98**, 144431 (2018).
46. Uchida, K., Nonaka, T., Kikkawa, T., Kajiwara, Y., Saitoh, E. Longitudinal spin Seebeck effect in various garnet ferrites. *Physics Review B* **87**, 104412 (2013).
47. Volta, A, Nuova memoria sull'elettricità animale del Sig. Don Alessandro Volta. *Annali di chimica e storia* **5**, 132-144 (1794).
48. Uchida, K., et al. Observation of the spin Seebeck effect. *Nature* **455**, 778-781 (2008).
49. Uchida, K., et al. Thermoelectric generation based on spin Seebeck effects. *Proceedings of the IEEE* **104**, 1946-1973 (2016).
50. Rezende, S. M., et al. Magnon spin-current theory for the longitudinal spin-Seebeck effect. *Physics Review B* **89**, 014416 (2014).
51. Kikkawa, T., et al. Longitudinal spin Seebeck effect free from the proximity Nernst effect. *Physics Review Letters* **110**, 067207 (2013).
52. Saitoh, E., Ueda, M. & Miyajima¸ H. Conversion of spin current into charge current at room temperature: Inverse spin-Hall effect, *Appl. Phys. Lett.* **88**, 182509 (2006).

53. Miao, B. F., Huang, S. Y., Qu, D. & Chien, C. L. Inverse spin Hall effect in a ferromagnetic metal, *Phys. Rev. Lett.* **111**, 066602 (2013).
54. d'Allivy Kelly, O., et al. Inverse spin Hall effect in nanometer-thick yttrium iron garnet/Pt system. *Appl. Phys. Lett.* **103**, 082408 (2013).
55. Tao, X., et al. Self-consistent determination of spin Hall angle and spin diffusion length in Pt and Pd: The role of the interface spin loss. *Science Advances* **4**, EAAT1670 (2018).
56. Guyer, J.E., Wheeler, D. & Warren J.A. FiPy: Partial Differential Equations with Python. *Computing in Science & Engineering* **11** (3), 6-15 (2009).
57. Geuzaine, C., Remacle, J-F. Gmsh: A 3-D finite element mesh generator with built-in pre- and post-processing facilities. *Numerical Methods in Engineering* **79** (11) 1309-1331 (2009).
58. Hall, E. H., On a New Action of the Magnet on Electric Currents. *American Journal of Mathematics* **2**, 287-292 (1879).
